# Regulation of interferon alpha production by the MAGUK-family protein CASK under H5N1 infection

**DOI:** 10.3389/fimmu.2024.1513713

**Published:** 2025-01-09

**Authors:** Jing-Ying Huang, Pei-Shan Sung, Shie-Liang Hsieh

**Affiliations:** ^1^ Doctoral Degree Program of Translational Medicine, National Yang Ming Chiao Tung University and Academia Sinica, Taipei, Taiwan; ^2^ Immunology Research Center, National Health Research Institute, Zhunan, Taiwan; ^3^ Institute of Clinical Medicine and Institute of Microbiology and Immunology, National Yang Ming Chiao Tung University, Taipei, Taiwan; ^4^ Department of Medical Research, Taipei Veterans General Hospital, Taipei, Taiwan; ^5^ Master Program in Clinical Genomics and Proteomics, School of Pharmacy, Taipei Medical University, Taipei, Taiwan

**Keywords:** CASK, H5N1, influenza A virus, macrophage, nuclear entry, interferon-alpha, CRM1, mRNA export

## Abstract

CASK, a MAGUK family scaffold protein, regulates gene expression as a transcription co-activator in neurons. However, the mechanism of CASK nucleus translocation and the regulatory function of CASK in myeloid cells remains unclear. Here, we investigated its role in H5N1-infected macrophages. We found that H5N1 triggers CASK nuclear translocation via PKR and SRC signaling. HCK, a SRC family kinase, enhances CASK phosphorylation at S395 via CDK5, facilitating CASK’s nuclear entry. Knocking out CASK in myeloid cells specifically reduces interferon-alpha (IFNA) production by hindering the nuclear export of *Ifna* mRNA, while leaving its mRNA levels unchanged. Myeloid-specific CASK knockout (KO) mice display exacerbated lung inflammation, which correlates with reduced IFNA levels during H5N1 infection. Interactome studies show that H5N1 triggers associations between CASK and CCT4, STIP1, and TNK1. These associations recruit IRF7, POLR2C, TAF15, HNRNPs, and CRM1, enabling the CASK complex to bind to the *Ifna* promoter, bind co-transcriptionally to *Ifna* mRNA, and facilitate CRM1-dependent *Ifna* mRNA export. This underscores CASK’s critical role in the antiviral response.

## Introduction

Calcium/calmodulin-dependent serine protein kinase (CASK) is a scaffold protein belonging to the membrane-associated guanylate kinases (MAGUKs) family (1). The human CASK protein, composed of 926 amino acids, includes a calmodulin domain, two L27 domains, one PDZ domain, one SH3 domain, and a guanylate kinase domain. These domains are known to function as protein–protein interaction modules, making CASK a key scaffold protein for assembling large signaling complexes (1). Previous studies have shown that CASK is expressed at both the pre- and postsynaptic sides of excitatory synapses and is crucial for cerebrocortical development. Mutations in CASK have been linked to mental retardation, autism-spectrum disorders (2, 3), epilepsy (4), cerebellar hypoplasia (5, 6), and X-linked intellectual disability (7). CASK has been shown to interacts with Tbr-1, a T-box transcription factor that is involved in forebrain development (8). Although CASK is primarily located in the juxta-membrane region of neuronal cells, co-transfection of CASK and Tbr-1 results in nuclear translocation and binding to specific DNA sequences in a complex with Tbr-1 to induce the transcription of T-element-containing genes (9), suggesting that CASK acts as a co-activator for gene transcription. However, the mechanism by which CASK translocates from the membrane to the nucleus remains unknown.

In addition to neurons, CASK is also expressed in other cell lineages, including immune and non-immune cells. However, the functions of CASK in non-neuronal cells are not yet well understood. CASK is abundantly expressed in macrophages and is secreted from macrophages via exosomes. Interestingly, CASK is detectable in the serum of patients with focal and segmental glomerulosclerosis (FSGS), a condition that causes nephrotic syndrome with a risk of progressing to end-stage renal disease. Moreover, recombinant CASK alters the permeability of a monolayer of podocytes and increases the motility of podocytes (10), suggesting that CASK, either alone or in the context of exosomes, may act as an endogenous danger signal to trigger inflammatory reactions (10).

Macrophages play a pivotal role in immune responses, serving as a key component of the host defense against pathogens such as the H5N1 influenza A virus (IAV). The H5N1 influenza A virus (IAV) can establish productive infections in macrophages (11), activating intracellular viral RNA sensors such as RIG-I (12, 13). These sensors initiate signaling cascades that induce the transcription of type I interferons (14, 15) (including interferon-beta and interferon-alpha) and pro-inflammatory cytokines (16). This activation results in a cytokine storm (17, 18), a characteristic double-edged phenomenon of H5N1 infection. While the cytokine response helps control viral spread, it simultaneously contributes to immunopathology, highlighting the complex interplay between host defense mechanisms and viral pathogenesis.

Previous studies have shown that CASK can translocate into the nucleus and function as a transcription co-activator (9), regulating gene expression in neurons. We hypothesize that CASK’s ability to regulate gene expression may be comparable to other scaffold proteins, such as MAVS (19) and NEMO (20), which are known to orchestrate antiviral signaling by serving as platforms for assembling signaling complexes. This hypothesis is supported by the modular domains of CASK that enable diverse protein-protein interactions, positioning it as a potential regulator of gene expression in response to cellular stress or infection. In this study, we aim to investigate whether CASK is involved in the signal transduction pathway regulating the H5N1-induced cytokine storm in macrophage, and if so, to elucidate the underlying molecular mechanisms.

Confocal fluorescence microscopy reveals that H5N1 infection triggers CASK nuclear translocation. Kinase inhibitor screening identifies PKR and SRC signaling as key regulators of CASK’s nuclear entry. Overexpression of the SRC-family kinase HCK and an EGFP-CASK fusion protein in 293T cells, followed by IP-LC-MS/MS analysis, shows a 10-fold increase in S395 phosphorylation. Conversely, the S395A mutation diminishes the HCK-induced nuclear EGFP signal. In H5N1-infected cells, CASK knockout selectively reduces interferon-alpha (*Ifna*) production without affecting its mRNA levels. RNA-FISH demonstrates a marked increase in the nuclear-to-cytoplasmic ratio of *Ifna* mRNA in CASK-deficient macrophages, indicating CASK’s role in *Ifna* mRNA nuclear export. *In vivo*, myeloid-specific CASK knockout mice exhibit more severe lung inflammation, correlating with significantly decreased lung IFNA levels. Interactome studies (IP-LC-MS/MS and NCBI database analysis) reveal enhanced associations between CASK and CCT4, STIP1, and TNK1 under H5N1 infection. These primary interactions recruit secondary interactors such as IRF7, POLR2C, HNRNPs, and CRM1, facilitating CASK complex binding to the *Ifna* promoter, co-transcriptional mRNA binding, and more efficient CRM1-dependent mRNA export. In summary, our study elucidates the mechanisms governing CASK nuclear translocation and CASK-mediated IFNA expression in response to H5N1 infection. This work provides crucial insights into the intricate interplay between viral infection, cellular signaling, and the host immune response, highlighting CASK’s pivotal role in orchestrating antiviral defenses.

## Results

### Upregulation and nucleus translocation of CASK in H5N1-IAV-infected macrophage

**Figure 1:**
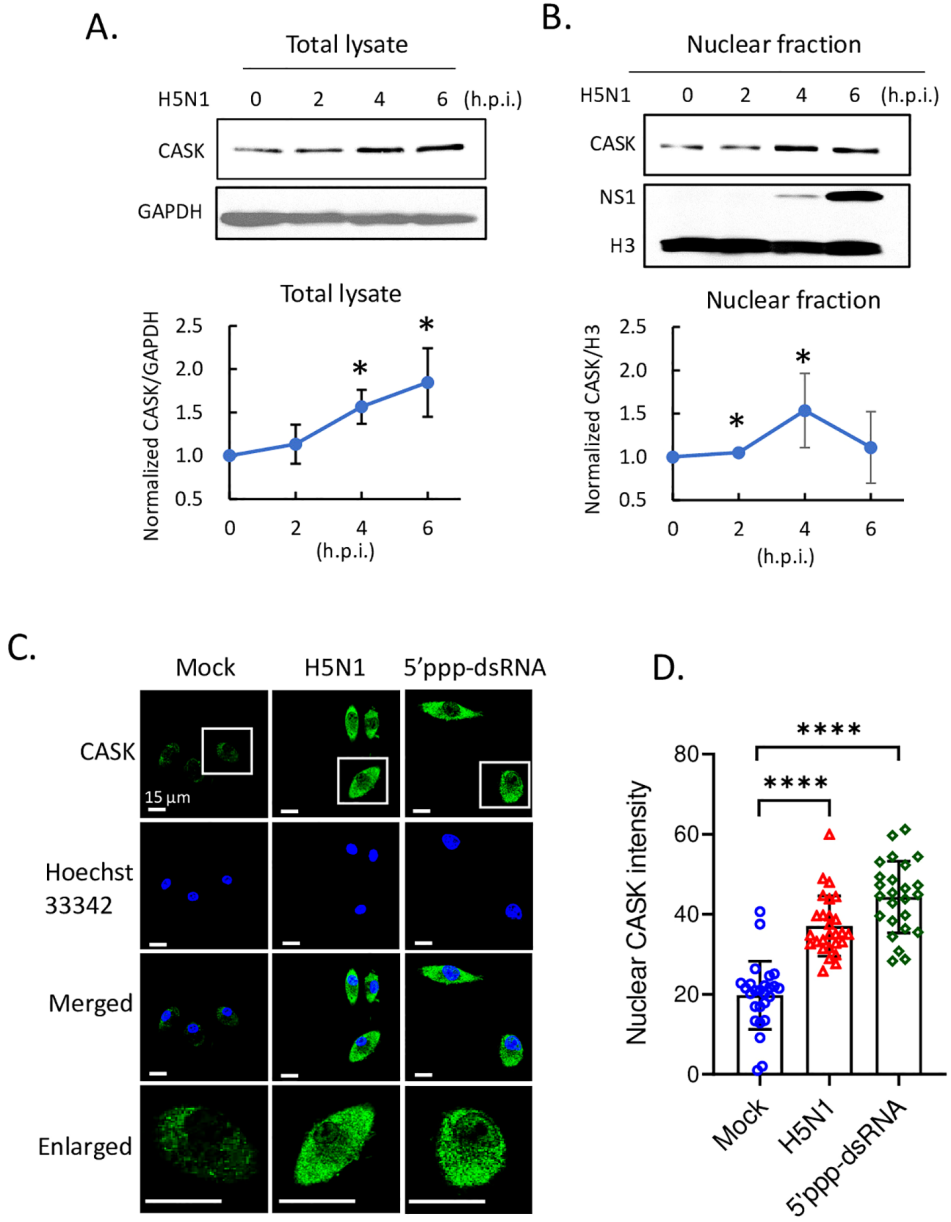
H5N1 infection and RIG-I ligand stimulation trigger CASK upregulation and nucleus entry. Mouse primary GM-MΦ infected with H5N1 MOI=1 for 1 hour and harvested at 0, 2, 4, 6 hours post infection (h.p.i.). **(A)** Total lysate analyzed by SDS-PAGE and western blot. **(B)** H5N1 infected primary GM-MΦ are fractionated into nuclear fraction and then analyzed by SDS-PAGE and western blot. H3, histone H3, nuclear marker. NS1, influenza a virus nonstructural protein 1. **(A, B)** Quantification data are from three independent experiments. **(C)** primary GM-MΦ are seeded onto coverslip in 24 well and infected with H5N1, MOI=1, or transfected with 5’ppp dsRNA 400 ng/ml, for 3 hours and then fixed with 4% paraformaldehyde (Electron Microscopy Sciences, 15710), stained with anti-CASK antibody, anti-mouse Alexa488 secondary antibody and Hoechst33342, and analyze nuclear CASK by confocal fluorescence microscope. **(D)** Nuclear CASK intensity is analyzed using LAS-X software. *p < 0.05, ****p < 0.0001 (Student’s t-test).

To investigate the roles of CASK in myeloid cells, we incubated mouse bone marrow-derived macrophages (BMDMs) with H5N1-IAV and examined CASK expression and subcellular localization. We observed that the overall expression of CASK increased in a time-dependent manner (**Figure 1A**). In contrast, nuclear CASK expression increased within the nucleus at 2 h and peaked at 4 h (**Figure 1B**). The cytosolic abundance of CASK is significantly higher than its nuclear counterpart, making direct side-by-side comparisons challenging. Therefore, we focused our analysis on the time course of CASK expression within the nuclear fraction. Evidence of macrophage infection by H5N1-IAV was confirmed by the expression of the non-structural protein NS1 starting from 4 h post-infection (**Figure 1B**). These observations indicate that H5N1-IAV infection upregulates CASK expression and triggers its translocation into the nucleus, starting at 2 h and peaking at 4 h post-infection. Immunofluorescence confocal microscopy analysis confirmed the upregulation and nuclear translocation of CASK (**Figure 1C**), with CASK intensity quantified in **Figure 1D**.

To elucidate how H5N1-IAV upregulates CASK expression and promotes nuclear translocation, BMDMs were incubated with various ligands capable of activating different nucleic acid receptors and sensors. Notably, transfected 5’ppp dsRNA (a ligand of RIG-I and PKR) and transfected poly (I:C) (a ligand of RIG-I/MDA-5) induced CASK upregulation and nuclear translocation similar to H5N1-IAV, whereas un-transfected poly (I:C) (a TLR3 ligand) and CL097 (a TLR-7/8 ligand) only increased CASK expression in the juxta-membrane region (**Supplementary Figure S1**).

### CASK deficiency impaired the production of IFNA in response to H5N1-IAV by disrupting the nuclear export of IFN-α mRNA

As systemic knockout of *Cask* is neonatal-lethal in mice (21), we generated mice with tissue-specific *Cask* knockout by introducing LoxP sites flanking *Cask* exon 2 on X chromosome (**Supplementary Figure S2A**). To achieve myeloid-specific knockout of Cask, we crossed heterozygous floxed females (*Cask* ^f/x^) with LysM-Cre male (**Supplementary Figure S2B**). As a result, the male offspring (*Cask* ^f/y^ LysM-Cre^+^) carried the myeloid-specific Cre, leading to the deletion of CASK in myeloid cells such as macrophages and granulocytes (22). We first confirmed the deletion of exon 2 of *Cask* in genomic DNA by PCR (**Supplementary Figure S2C, D**) in macrophages from *Cask* ^f/y^ LysM-Cre^+^ mice. SDS-PAGE and western blot analysis further validated the absence of CASK protein in these CASK-deficient macrophages (**Supplementary Figure S2E**).

**Figure 2:**
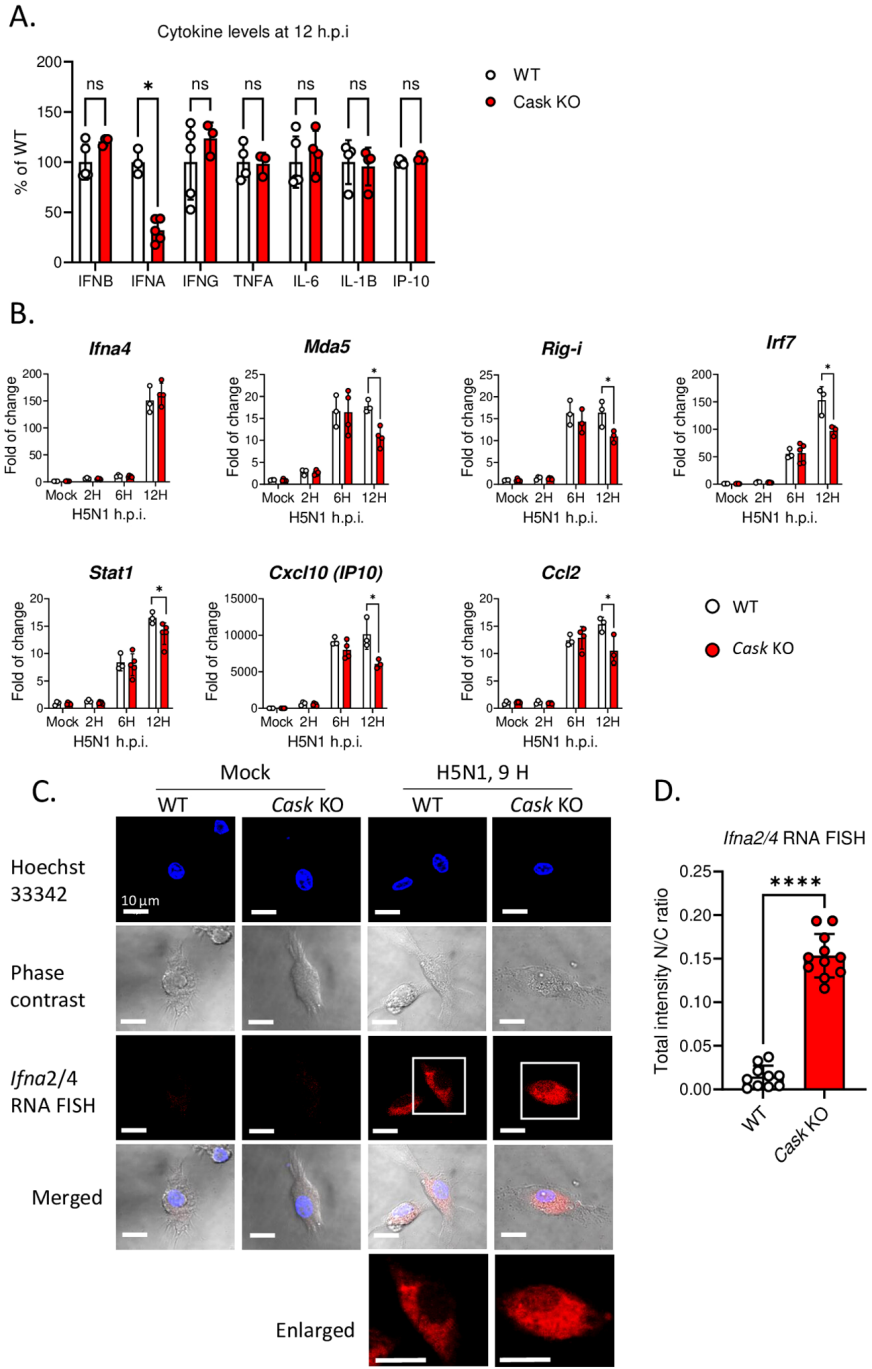
CASK deficiency selectively blunts H5N1-induced IFNA production through perturbation of Ifna mRNA export. **(A)** Primary GM-MΦ derived from CASK wild type mouse (WT=*Cask* ^wt^LysMcre+) and myeloid-specific CASK deficient mouse (*Cask* KO=*Cask* ^flox^LysMcre+) were infected with H5N1, MOI=1, for 12 hours, and the culture supernatant were collected and subjected to ELISA analysis of IFNB, IFNA, IFNG, TNFA, IL6, IL-1B, and IP-10. **(B)** Primary GM-MΦ derived from WT and *Cask* KO were infected with H5N1, MOI=1, for 2, 6, 12 hours, RNA were extracted for RT-qPCR to evaluate mRNA levels of *Ifna4* and interferon-stimulated genes: *Mda-5, Rig-I, Irf7, Stat1, Ip-10* and *Ccl2.* **(C)** Primary GM-MΦ derived from WT and *Cask* KO were seeded onto coverslip and infected with H5N1, MOI=1, for 9 hours, cells were then fixed, stained with Hoechst 33342 and hybridize with fluorescent oligonucleotide probes specifically recognize *Ifna2* and *Ifna4* mRNA sequences. This RNA FISH (fluorescence *in situ* hybridization) samples were then analyzed by confocal fluorescence microscope. Nucleus and cytoplasm fluorescence intensity were analyzed by MetaMorph software. Total intensity N/C ratio= (fluorescence integrated intensity of nucleus)/(fluorescence integrated intensity of cytoplasm) of each cell were calculated and shown in **(D)**. Scale bar=10 μm. *p < 0.05, ****p < 0.0001 (Student’s t-test).

Upon H5N1-IAV infection, macrophages secrete interferon alpha (IFN-α) and pro-inflammatory cytokines (16, 23). To investigate the impact of CASK on cytokine production, we measured the levels of IFN-β, IFN-α, IFN-6, TNF-α, IL-6, IL-1β and IP-10 in the culture supernatant of macrophages at 12 h post-H5N1-IAV (MOI=1) infection. Surprisingly, we observed a 68% reduction in IFN-α in CASK-deficient GM-MΦ, while the levels of other 6 cytokines (IFNB, IFNG, TNFA, IL-6, IL1B, IP-10) remained unaffected (**Figure 2A**). Despite the 68% decrease in protein expression, the mRNA level of *Ifna4* remained unchanged when comparing WT and *Cask* KO macrophages (**Figure 2B**). Consistent with the decreased IFN-α level at 12 hours post-infection, the mRNA levels of various interferon-stimulated genes (ISGs) were significantly reduced in *Cask* knockout (KO) macrophages. The extent of transcriptional induction of these ISGs, including *Mda5, Rig-I, Irf7, Stat1, Cxcl10*, and *Ccl2*, reflects the signal intensity of type I interferons. In contrast, the mRNA levels of other interferons (*Ifnb1, Ifnl2 and Ifng*), proinflammatory cytokines (*Il1b, Il18*), *Pkr, Tlr7* and the inflammasome gene *Nlrp3* remained unaffected when comparing WT and *Cask* KO macrophages (**Supplementary Figure S3**). In addition, CASK-deficiency does not affect H5N1 IAV viral gene expression (NP and M, **Supplementary Figure S3L, M**) or viral titer (**Supplementary Figure S3N**).

To understand how CASK-deficiency reduces IFN-α secretion without affecting its transcription, we first ruled out the possibility of CASK involvement in the IFN-α secretory pathway. Our findings revealed no intracellular accumulation of IFN-α in CASK-deficient macrophages following IAV infection (**Supplementary Figure S4**). We then investigated whether the nuclear export of *Ifna* mRNA was impaired in CASK-deficient macrophages. Using fluorescence *in situ* hybridization (RNA-FISH), we observed that most *Ifna* mRNA was efficiently exported to the cytosol in wild type macrophage infected with H5N1. In contrast, in CASK-deficient macrophages, a higher percentage of *Ifna*2/4 mRNA remained in the nucleus (nucleus/cytosol ratio: 0.15) at 9 h post H5N1-IAV infection in the CASK-deficient macrophages (**Figures 2C, D**). This suggests that H5N1-IAV induces CASK translocation into the nucleus to facilitate the export of *Ifna* mRNA to the cytosol.

### Regulation of CASK expression and nuclear translocation by PKR and Src kinases

**Figure 3:**
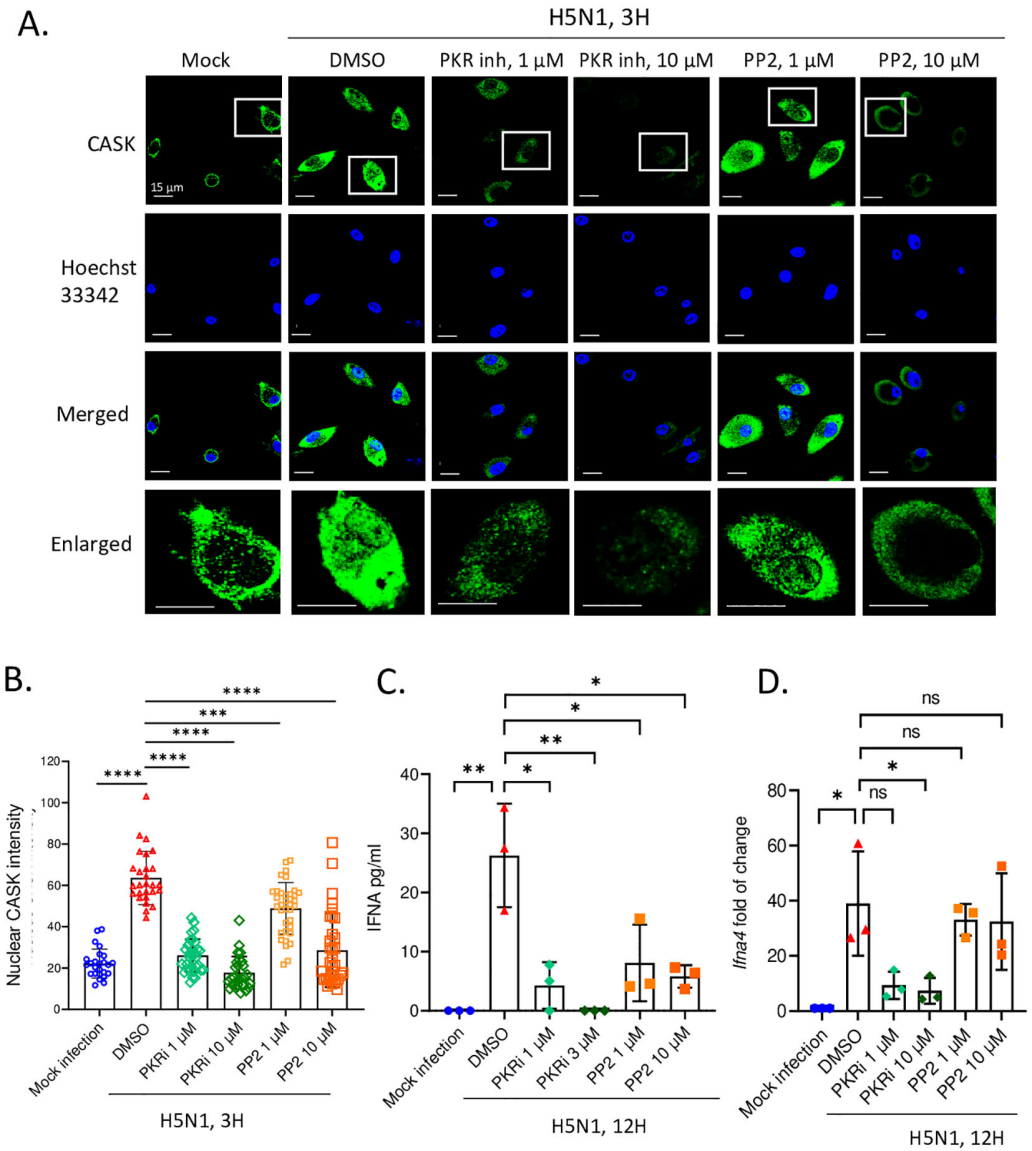
PKR and SRC inhibitors suppress H5N1-induced CASK nuclear translocation and repress IFNA production at transcriptional and post transcriptional levels, respectively. **(A-D)** Primary GM-MΦ are the attached cells derived from freshly isolated mouse bone marrow cells cultured for 7 days with 10 ng/mL mouse GM-CSF. H5N1 infection was performed in serum-free RPMI medium for 1 hour at 37°C, MOI=1. **(A)** Primary GM-MΦ are seeded onto coverslip in 24-well overnight and infected with H5N1, MOI=1, for 3 hours and then fixed with 4% paraformaldehyde (Electron Microscopy Sciences, 15710), stained with anti-CASK antibody, anti-mouse Alexa488 antibody and Hoechst 33342, then analyze nuclear translocation of CASK by confocal fluorescence microscope. Scale bar=15 μm. **(A)** Inhibitors are diluted to the indicated concentrations in RPMI 10% serum culture medium and incubate with H5N1 infected GM-Mf for 3 hours. **(B)** Quantification of nucleus intensity by LAS-X software to reflect the effects of inhibitors on CASK nucleus translocation. **(C, D)** H5N1-infected Primary GM-MΦ were treated with indicated kinase inhibitors for 12 hours and culture supernatants were analyzed by IFNA2/4 ELISA **(C)**; cells were harvested with Trizol at 12-hours post-infection and total RNA were extracted. *Ifna4* mRNA levels were analyzed by RT-qPCR **(D)**. *p < 0.05, **p < 0.01, ***p < 0.001, ****p < 0.0001 (Student’s t-test).

We further investigated the signaling pathway responsible for the upregulation and nuclear translocation of CASK. Given the crucial role of PKR in antiviral responses (24) and the activation of Src family kinases (SFKs) by influenza virus (25), we examined whether PKR and Src family kinases contributed to the elevation and nuclear translocation of CASK following H5N1-IAV infection. Using confocal microscopy-based screening with various kinase inhibitors (unpublished data), we found that PKR inhibition most effectively suppressed CASK upregulation (**Figure 3A**). The Src inhibitor (PP2) successfully blocked CASK nuclear translocation. The quantification of nuclear CASK is shown in **Figure 3B**.

Of particular interest is the observation that PKR inhibition not only suppressed the production of IFN-α but also its transcription (**Figures 3C, D**), while the Src inhibitor (PP2) selectively impeded IFN-α translation without affecting its transcription (**Figures 3C, D**). These data suggest that the Src kinase inhibitor specifically restrains CASK nuclear trans location and IFN-α secretion without interfering with transcription. Importantly, these findings demonstrate a strong positive correlation between the extent of CASK nuclear translocation and the abundance of secreted IFN-α, underscoring the critical role of CASK in modulating the antiviral response.

### HCK contributes to CASK nucleus translocation during H5N1-IAV infection

**Figure 4:**
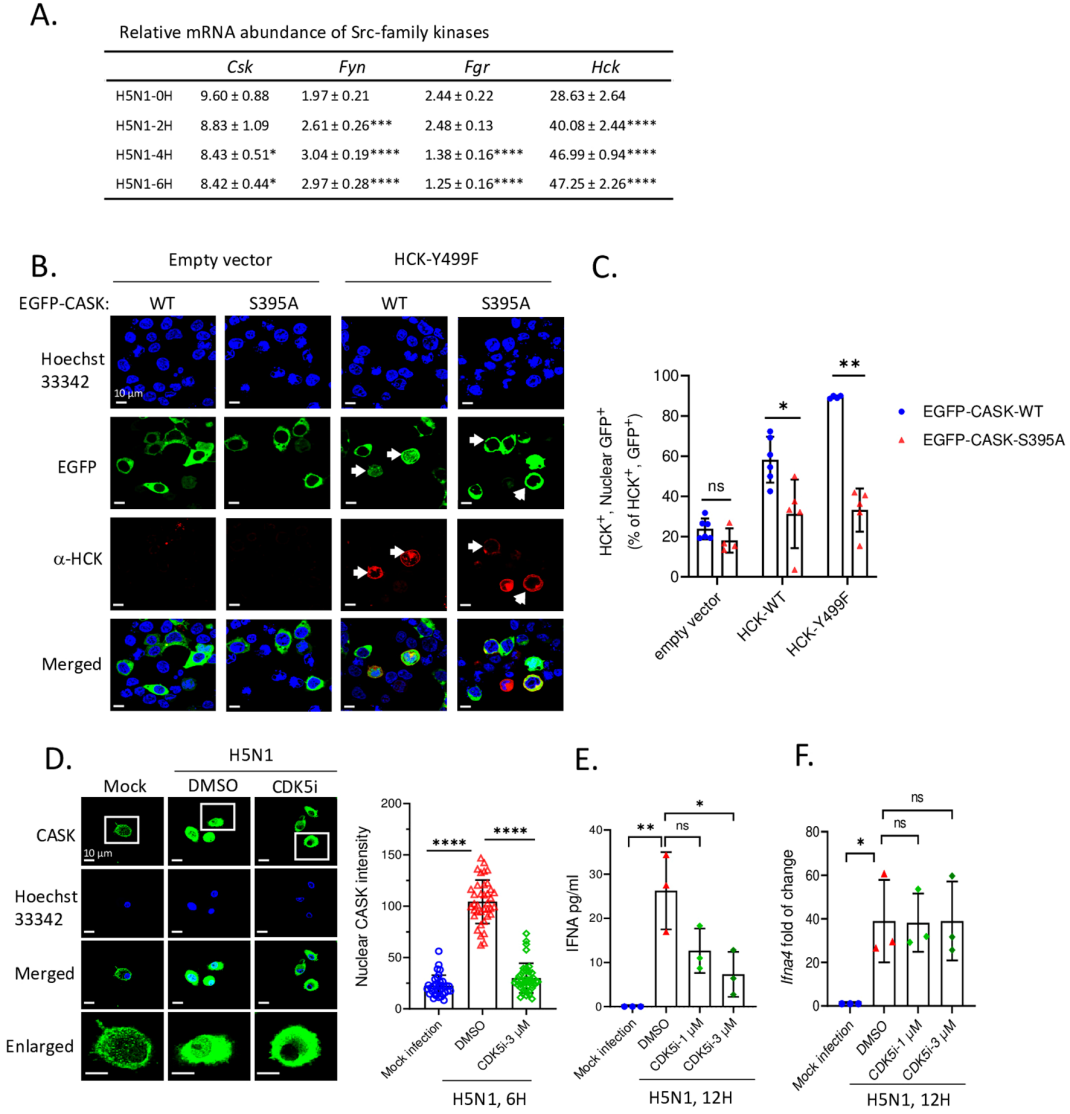
HCK regulates CASK S395 phosphorylation and nuclear translocation via CDK5 during H5N1 infection. **(A)** There are 4 out of 9 Src-family kinase members express in myeloid cells. Its abundance in H5N1-infected GM-MΦ are quantified by RT-qPCR. **(B)** 293T cells were seeded onto coverslip and transfected with EGFP-CASK (either wild-type or S395A) and co-overexpressed with either pCMV-empty vector, pCMV-HCK-WT or pCMV-HCK-Y499F. 24 hours after transfection, cells were fixed and stained with anti-HCK antibody, anti-mouse APC conjugated antibody and Hoechst 33342, then analyzed with fluorescence confocal microscope. HCK-expressing cells are counted as either nuclear GFP positive or negative from 15 fields for each treatment group and the statistical analysis is shown in **(C)**. **(D)** Primary GM-MΦ were mock infected or infected with H5N1, MOI=1. Cells were seeded onto coverslip and treated with DMSO or CDK5 inhibitor 3 μM for 6 hours and then fixed, stained and analyzed by fluorescence confocal microscope. Nuclear CASK intensity from the images acquired in **(D)** is calculated by Las X software. **(E, F)** H5N1-infected GM-MΦ were treated with CDK5 inhibitor for 12 hours and culture supernatant were analyzed by IFNA2/4 ELISA **(E)**; cells were lyzed with Trizol, total RNA were extracted and Ifna4 mRNA levels are analyzed by RT-qPCR **(F)**. *p < 0.05, **p < 0.01, ***p < 0.001, ****p < 0.0001 (Student’s t-test).

Since the SRC inhibitor PP2 is a general inhibitor for Src family kinases (SFKs), which includes nine members (Csk, Yes, Fyn, Fgr, Lck, Hck, Blk, Lyn, and Frk), we aimed to identify the specific member responsible for the nuclear translocation of CASK. First, we examined the expression of SFKs in macrophages (Csk, Fyn, Fgr, and Hck) following H5N1-IAV infection (**Figure 4A**). This observation suggests that Hck is the most abundant SFK and may contribute to virus-induced nuclear translocation of CASK. To test this, we generated constitutively active forms of HCK: HCKΔ513-524 (deletion mutant) and HCK Y499F mutant (Y→F mutation) (26), then co-transfected them with an EGFP-CASK construct into 293T cells. Immunoprecipitation with anti-GFP mAb, followed by trypsin digestion and LC-MS/MS analysis, revealed that compared to the control group, the phospho/nonphospho ratio at residue Serine 395 (S395) of CASK increased approximately 10-fold (0.008 to 0.072 by HCKΔ513-524, 0.008 to 0.108 by HCK Y499F), while the other 8 potential phosphorylation sites did not show significant alterations (**Table 1**).

**Table 1:** Identification of HCK-dependent CASK phosphorylation site S395 by IP-LC-MS-MS.

Phospho-peptide sequences	Phospho-site	Phospho/Nonphospho ratio		
		EV^a^	HCK512^b^	HCK-Y499F^c^
LMLDPAERITVY*EALNHPWL	Y268	0.000	0.014	0.296
YDKINTKSS*PQIRNPPSDAVQRA	S395	0.008	0.072	0.108
RILT*QPHFMA	T433	0.006	0.003	0.009
MALLQTHDVVAHEVY*SDEALRVTPPPTSPYL	Y452	0.560	0.837	0.479
LRVT*PPPTSPYL	T460	0.050	0.036	0.055
YSDEALRVTPPPTS*PYL	S465	0.268	0.161	0.175
YRT*QSSSCERDSPSTSRQ	T573	0.012	0.000	0.000
LPST*TQPKGRQ	T607	0.000	0.033	0.015
RQIY*VRA	Y616	0.029	0.034	0.098

*Phosphorylation; ^a^EV, empty vector; ^b^HCK512, HCKΔ513-524, constitutively active form of HCK.; ^c^HCK-Y499F, constitutively active form of HCK.

Constitutively active HCK are co-overexpressed with EGFP-CASK in 293T cells. The cell lysate were then subjected to immunoprecipitation using anti-GFP antibody. The IP products were subjected to SDS-PAGE analysis and the protein band corresponding to EGFP-CASK (130 KD) were cut and analyzed by mass spectrometry LC-MS-MS. 9 phospho-peptides were identified and the one containing phospho-S395 showed constitutively-active-HCK-dependent 10 fold increase comparing to co-overexpression with empty vector (EV). Notably, this serine phosphorylation site indicates one or more serine-threonine kinases were activated by HCK, which is a tyrosine kinase. Later on, we use NetPhos-3.1 to predict which kinase could phosphorylate CASK at S395 site.

To confirm the phosphorylation of S395 of CASK is required for nuclear translocation, we generated an EGFP-CASK S395A mutant (S→A mutation) to prevent CASK phosphorylation. In the absence of HCK Y499F, both the wild type (CASK S395) and mutant (CASK S395A) remained in the cytosol (left two columns, **Figure 4B**). In contrast, transfection of HCK Y499F (red color) induced the translocation of wild type CASK (green color) into the nucleus (3^rd^ column from left, **Figure 4B**), an effect not observed with the CASK S395A mutant (4^th^ column from left, **Figure 4B**). The percentage of nuclear GFP-CASK (60% and 90% in WT and 35% in S395A) resulting from the overexpression of wild type HCK and HCK Y499F is shown in **Figure 4C**. Thus, we concluded that activated HCK induces the phosphorylation of CASK at Ser_395_, facilitating its nuclear translocation.

Since HCK, a tyrosine kinase, is unlikely to directly phosphorylate S395 of CASK, we used the NetPhos-3.1 software (27) to predict the potential serine-threonine kinase that could phosphorylate CASK at S395. Our analysis revealed that cyclin-dependent kinase 5 (CDK5) (**Table 2**) is the most likely candidate kinase downstream to HCK. This finding aligns with previous reports showing that CDK5 phosphorylates CASK at S395 in neurons (28). To confirm the involvement of CDK5 in CASK nuclear translocation, we incubated macrophage with H5N1-IAV in the presence of a CDK5 inhibitor. While CASK upregulation remained unaffected, the H5N1-IAV-induced nuclear translocation of CASK (Nuclear CASK intensity average: 105) was suppressed by the CDK5 inhibitor (Nuclear CASK intensity average: 26) (**Figure 4D**). Thus, we concluded that H5N1-IAV infection activates HCK to phosphorylate CASK at residue S395 via CDK5, thereby inducing the nuclear translocation of CASK. Moreover, the CDK5 inhibitor also decreased H5N1-induced IFN-α production (from average 27 pg/ml in DMSO treatment to average 7 in CDK5 inhibitor 3 µM treatment) (**Figure 4E**) without suppressing *Ifna* mRNA levels (**Figure 4F**). These observations further confirm the crucial role of the HCK-CDK5 axis in regulating CASK nuclear translocation during H5N1-IAV infection.

**Table 2:** Prediction of upstream kinases phosphorylating CASK at S395 and CDK5 at Y15 by NetPhos-3.1.

Protein	PTM site	Context seq.	Score	Kinase	Answer
CASK	395S	NTKSSPQIR	0.501	CDK5	Yes
CASK	395S	NTKSSPQIR	0.461	GSK3	No
CASK	395S	NTKSSPQIR	0.436	P38MAPK	No
CASK	395S	NTKSSPQIR	0.419	CaM-II	No
CASK	395S	NTKSSPQIR	0.386	CDC2	No
CASK	395S	NTKSSPQIR	0.359	CKI	No
CASK	395S	NTKSSPQIR	0.351	DNAPK	No
CASK	395S	NTKSSPQIR	0.331	RSK	No
CASK	395S	NTKSSPQIR	0.300	PKG	No
CASK	395S	NTKSSPQIR	0.289	ATM	No
CASK	395S	NTKSSPQIR	0.255	CKII	No
CASK	395S	NTKSSPQIR	0.185	PKA	No
CASK	395S	NTKSSPQIR	0.107	UNSP	No
CASK	395S	NTKSSPQIR	0.087	PKB	No
CASK	395S	NTKSSPQIR	0.077	PKC	No
CDK5	15Y	GEGTYGTVF	0.611	UNSP	YES
CDK5	15Y	GEGTYGTVF	0.591	SRC	YES
CDK5	15Y	GEGTYGTVF	0.545	EGFR	YES
CDK5	15Y	GEGTYGTVF	0.495	INSR	No

1. CASK, peptide sequence: NP_001271432.1, 926 amino acids.

2. CDK5, peptide sequence: XP_006535689.1, 292 amino acids.

The amino acid sequence of CASK (NP_001271432.1) in FASTA format was submitted to the NetPhos-3.1 online software for upstream kinase prediction. Cdk5, with a score ≥ 0.5, was predicted to be the upstream kinase responsible for phosphorylating CASK at S395. The amino acid sequence of CDK5 (NP_031694.1) in FASTA format was submitted to NetPhos-3.1 for upstream kinase prediction. SRC, with a score ≥ 0.5, was predicted as the upstream kinase for CDK5 Y15 phosphorylation.

### Myeloid-specific CASK knockout in mice reduce IFN-α production and exacerbate lung inflammation during H5N1-IAV LD50 challenge

**Figure 5:**
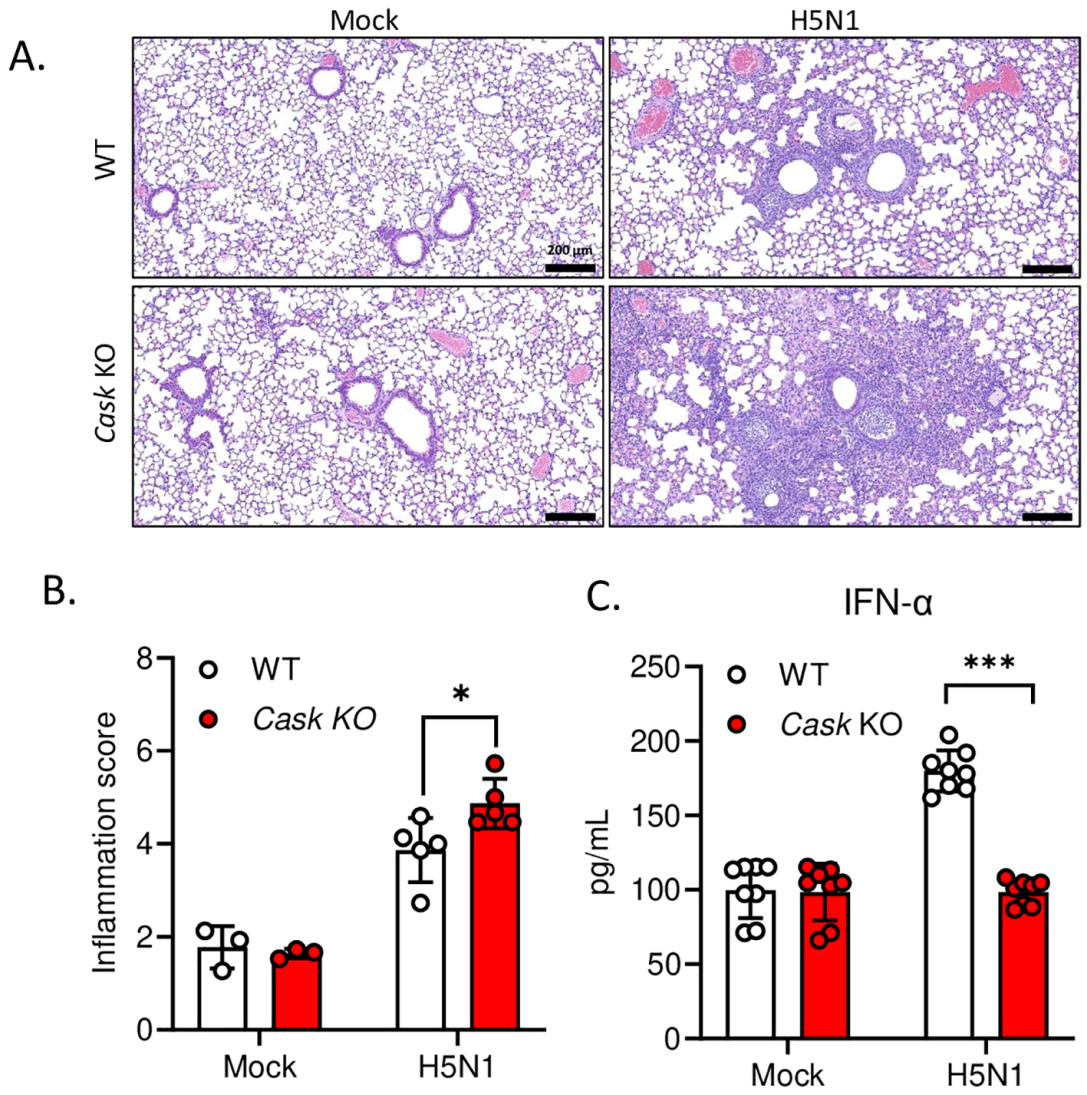
Myeloid-specific CASK knockout in mice reduce IFNA production and exacerbate lung inflammation during H5N1 LD50 challenge. *Cask* ^wt^LysMCre+ (WT) and *Cask^flox^* LysMcre+(*Cask* KO) mice were infected with H5N1 by intranasal challenge of LD50 (750 pfu/mouse), and sacrificed at post-infection day 3 **(C)** or day 5 **(A, B)**. **(A)** Haematoxylin and eosin staining of left lung cross section. Left lung histopathology shows peri-bronchial leukocyte infiltration in H5N1 infected groups. **(B)** The severity of lung inflammation in response to H5N1 infection at post-infection day 5 were quantified by inflammation score. The inflammation score evaluates two parts, peri-bronchial infiltration (0-4 points, 0, none; 1, mild, infiltrate ≤4 cells thick; 2, moderate, infiltrate 5-10 cells thick; 3, severe, infiltrate 20%-50% visualized lumens; 4, diffuse, infiltrate >50% visualized lumens), and alveolar involvement (0-3 points, 0, none; 1, mild, patchy increased cellularity/thickening; 2, moderate, 25%-50% visualized lung with increased cellularity/thickening; 3, severe, > 50% visualized lung with increased cellularity/thickening). Lung section scoring was performed at 4X magnification and divided one whole section into 15 fields. The inflammation scores of 15 fields were averaged to represent an overall score from one mouse (n=3-5). **(C)** Left lungs were collected at post infection day 3 and lung homogenates were analyzed by IFNA2/4 ELISA. *p < 0.05, ***p < 0.001 (Student’s t-test).

To assess the impact of CASK deficiency on attenuating H5N1-IAV-induced IFN-α production *in vivo*, we intranasally challenged myeloid-specific *Cask* knockout mice with a median lethal dose (LD50) of H5N1-IAV and then examined lung IFN-α production and histopathology. We observed significantly higher peri-bronchial leukocyte infiltration, alveolar cellularity, and thickening in the lungs of CASK-deficient mice compared to wild-type mice at day 5 post-infection (**Figures 5A, B**). The extent of H5N1-IAV-induced lung immunopathology in CASK-deficient mice corresponds with the reduced IFN-α production in the lungs (**Figure 5C**). These findings suggest that CASK plays a crucial role in regulating IFN-α production and contributes to the host’s defense against viral infection.

### IP-LC-MS/MS and meta-analysis of NCBI database reveal CASK interactants involving in processing of mRNA and CRM1-mediated mRNP nuclear export

We then investigated how nuclear CASK regulates the export of *Ifna* mRNA to the cytosol. As CASK is a scaffold protein known to interact with many proteins via its PDZ, L27, SH3, and GK domain (1), we explored whether CASK interacts with nuclear proteins involved in the export of messenger ribonucleoprotein (mRNP) to the cytosol. To address this question, macrophages were incubated with H5N1-IAV for 3 h, followed by a 2-step ultracentrifugation process to harvest cytosolic and nuclear fractions. The nuclear fractions were then incubated with Dynabeads^®^ coupled with anti-CASK antibody or an isotype control. Nuclear proteins associated with endogenous CASK were pulled down by the ani-CASK antibody-coupled Dynabeads^®^ then subjected to in-solution trypsin digestion, and analyzed via liquid chromatography-coupled tandem mass spectrometry (LC-MS/MS).

**Figure 6:**
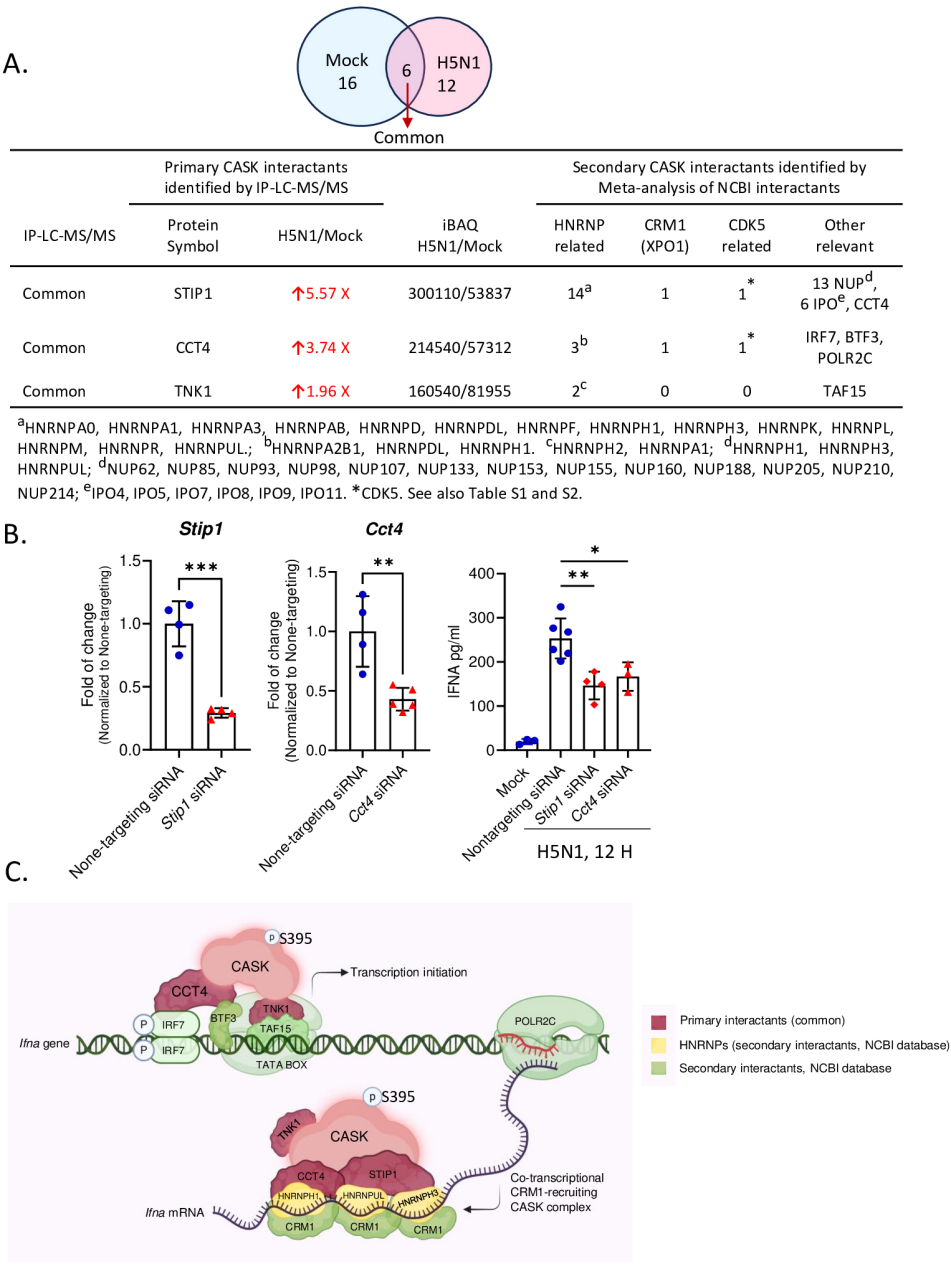
IP-LC-MS/MS analysis of H5N1-infected GM-MΦ and meta-analysis unveiled CASK-interacting proteins involving in mRNA processing and CRM1-mediated mRNA nuclear export. **(A)** Immunoprecipitation coupled LC-MS/MS analysis of nuclear fractions from H5N1-infected GM-MΦ (MOI=1, 6 hours post-infection) using anti-CASK antibody-conjugated Dynabeads identified CASK-interacting proteins upregulated upon infection. Subsequent NCBI interaction analysis prioritized CASK interactants involved in mRNA export (HNRNPs and CRM1), CASK regulating kinases (CDK5-related), and other relevant proteins, facilitating mechanistic modeling of CASK’s role in anti-viral responses. **(B)** Silencing of Cct4 and Stip1 suppressed H5N1-induced IFNA production in GM-MΦ. GM-MΦ were transfected with siRNAs targeting Cct4 and Stip1 for 48 hours prior to H5N1 infection (MOI=1) for 12 hours. Knockdown efficiencies were confirmed by RT-qPCR analysis of Cct4 and Stip1 mRNA levels in total RNA extracts. Culture supernatants were collected for measurement of IFNA protein levels by ELISA. **(C)** The proposed model elucidates how the nuclear CASK complex regulates IFNA mRNA nuclear export in GM-MΦ upon H5N1 infection. Following infection, IRF7 undergoes phosphorylation, translocate into the nucleus, and bind to IFNA gene promoter to initiate transcription. Concurrently, S395 phosphorylated CASK enters the nucleus and interacts with CCT4, STIP1, and TNK1. Through CCT4’s interaction with IRF7 and TNK1’s with TATA-box binding protein TAF15, the CASK complex is recruited to IFNA promoters. Subsequently, via CCT4’s interaction with POLR2C, the CASK complex tracks along with the transcribing IFNA mRNA. The CASK complex then, facilitated by CCT4 and STIP1, binds the nascent IFNA mRNA through RNA-binding HNRNPs and recruits the nuclear export factor CRM1 onto the transcript. This *Ifna* mRNA-coupled CASK complex could counteract the competition posed by influenza vRNP for CRM1-mediated nuclear export. *p < 0.05, **p < 0.01, ***p < 0.001 (Student’s t-test).

Following H5N1-IAV infection, we observed increased associations between CASK and STIP1 (5.57-fold), CCT4 (3.74-fold), and TNK1 (1.96-fold) (**Figure 6A**). Additionally, we identified potential associated proteins (secondary interactants) linked with STIP1, CCT4, and TNK1 (**Figure 6A**, **Table 3**) using data from the NCBI database. We also identified 11 nuclear proteins specifically associated with CASK post-H5N1-IAV infection (**Table 4**). The interactome study using CASK IP-LC-MS/MS (**Figure 6A**, **Table 4**) and meta-analysis of NCBI/Gene/interactions (**Table 3**) suggest that pre-existing nuclear CASK forms a complex with primary interactants (STIP1, CCT4, TNK1) in macrophages before stimulation. As CASK is phosphorylated at residue S395, these observations suggest that phosphorylated CASK (pCASK-S395) enters the nucleus and recruits more STIP1, CCT4, and TNK1 to form a complex. Consistent with the effect of CASK deficiency on IFN-α, siRNA-mediated knockdown of STIP1 and CCT4 significantly reduces IFN-α production by around 40% (**Figure 6B**).

**Table 3:** Selected NCBI interactants of IP-LC-MS-MS-identified CASK interactants.

CASK interactants (primary)	NCBI interactants (secondary)	Function (some are quoted from NCBI Gene Summary)
STIP1 Stress-induced-phosphoprotein 1	HNRNP heterogeneous nuclear ribonucleoprotein	“HNRNP proteins are associated with pre-mRNAs in the nucleus and influence pre-mRNA processing and other aspects of mRNA metabolism and transport.”
	CRM1 (XPO1) exportin 1	XPO1 mediates the nuclear export of both IFNA1 mRNA and Influenza A virus vRNP.
	CDK5 Cyclin-dependent kinase 5	CDK5 phosphorylates CASK on S395 and promote CASK nucleus entry upon H5N1 infection in GM-macrophage
	NUP nucleoporin	NUPs are the main components of the nuclear pore complex in eukaryotic cells.
	IPO importin	IPO is predicted to enable nuclear import signal receptor activity and nuclear localization sequence binding activity.
	CCT4 T-complex protein 1 subunit delta	In this study, CCT4 is proven to be a CASK interactant whose association with CASK is increased 3.7- fold upon H5N1 infection in GM-macrophage.
CCT4 T-complex protein 1 subunit delta	HNRNP heterogeneous nuclear ribonucleoprotein	“HNRNP proteins are associated with pre-mRNAs in the nucleus and influence pre-mRNA processing and other aspects of mRNA metabolism and transport.”
	CRM1 (XPO1) exportin 1	XPO1 mediates the nuclear export of both IFNA1 mRNA and influenza a virus vRNP.
	CDK5 Cyclin-dependent kinase 5	In this study, CDK5 phosphorylates CASK on S395 and promote CASK nucleus entry upon H5N1 infection
	IRF7 interferon regulatory factor 7	“IRF7 play a role in the transcriptional activation of virus-inducible cellular genes, including interferon alpha. IRF7 has a critical role in the innate immune response against DNA and RNA viruses.”
	BTF3 basic transcription factor 3	“BTF3 forms a stable complex with RNA polymerase IIB and is required for transcriptional initiation.”
	POLR2C RNA polymerase II subunit C	“POLR2C is the third largest subunit of RNA polymerase II, the polymerase responsible for synthesizing messenger RNA in eukaryotes.”
TNK1 Non-receptor tyrosine-protein kinase	HNRNP heterogeneous nuclear ribonucleoprotein	“HNRNP proteins are associated with pre-mRNAs in the nucleus and influence pre-mRNA processing and other aspects of mRNA metabolism and transport.”
	TAF15 TATA-box binding protein associated factor 15	“TAF15 plays a role in RNA polymerase II gene transcription as a component of a distinct subset of multi-subunit transcription initiation factor TFIID complexes.”

**Table 4:** CASK interactome identified by IP-LC-MS/MS.

CASK Interactome	Primary interactants	Gene Name
Common	Stip1	Stress-induced-phosphoprotein 1
	Cct4	T-complex protein 1 subunit delta
	Tnk1	Non-receptor tyrosine-protein kinase TNK1
	Stxbp5	Syntaxin-binding protein 5
	Cdca7l	Cell division cycle-associated 7-like protein
	Zfyve16	Zinc finger FYVE domain-containing protein 16
H5N1-specific	Ltb	Lymphotoxin-beta
	Olfr1105	Olfactory receptor
	Tap1	Antigen peptide transporter 1
	Atp6v1g1	V-type proton ATPase subunit G 1
	Hyal3	Hyaluronidase;Hyaluronidase-3
	Spen	Msx2-interacting protein
	Ptprb	Receptor-type tyrosine-protein phosphatase beta
	5830473C10Rik	albumin superfamily member 1
	Efhd2	EF-hand domain-containing protein D2
	Rnasel	2-5A-dependent ribonuclease
	Cntn2	Contactin-2

**Figure 7:**
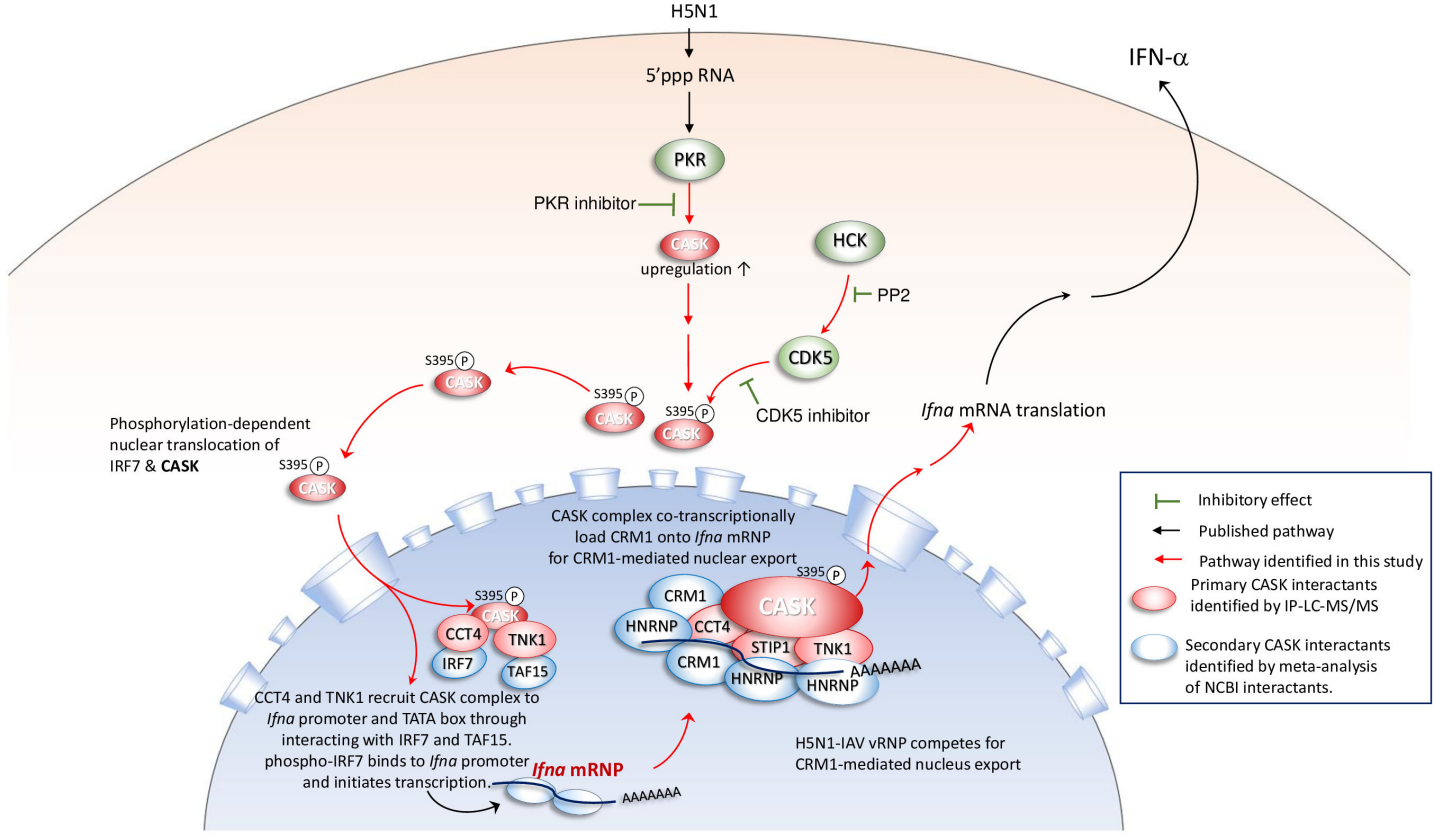
Phosphorylated CASK enters nucleus and forms a complex to facilitate CRM1-mediated *Ifna* mRNP export during H5N1 infection When the H5N1 virus infects macrophages, PKR senses the 5’ tri-phosphate group of the viral genomic RNA, activating a signaling pathway that induces the expression of IFN-α. PKR activity is crucial for the transcriptional activation of *Ifna*, as treatment with a PKR inhibitor suppresses both CASK and IFN-α expression. During H5N1 infection, the expression and activity of HCK are upregulated, positively correlating with CDK5-mediated phosphorylation of CASK at S395. The S395-phosphorylated CASK translocates to the nucleus, where it forms a complex with STIP1, CCT4, and TNK1, as confirmed by IP-LC-MS/MS analysis. This CASK complex is recruited to the *Ifna* promoters through CCT4’s interaction with IRF7. Additionally, the complex associates with nascent *Ifna* mRNA via secondary interactions with HNRNPs and facilitates CRM1 recruitment to the transcript, enabling CRM1-mediated nuclear export. This *Ifna* mRNA-coupled CASK complex may counteract the competitive binding of influenza vRNP to CRM1, thereby supporting efficient nuclear export of *Ifna* mRNA.

Based on these findings, we propose a mechanism model explaining how the CASK complex facilitates the nuclear export of *Ifna* mRNA (**Figure 6C**). The model elucidates the process by which the nuclear CASK complex regulates *Ifna* mRNA nuclear export in GM-MΦ upon H5N1 infection. Following viral infection, IRF7 undergoes phosphorylation, translocates to the nucleus, and binds to the *Ifna* gene promoter, initiating transcription. Simultaneously, CASK, phosphorylated at S395, enters the nucleus and interacts with CCT4, STIP1, and TNK1. The CASK complex is recruited to *Ifna* promoters through CCT4’s interaction with IRF7 and TNK1’s association with the TATA-box binding protein TAF15. Subsequently, via CCT4’s interaction with POLR2C, the CASK complex tracks along with the transcribing *Ifna* mRNA. Facilitated by CCT4 and STIP1, the CASK complex binds to the nascent *Ifna* mRNA through RNA-binding HNRNPs and recruits the nuclear export factor CRM1 onto the transcript (29). This *Ifna* mRNA-coupled CASK complex potentially counteracts the competition posed by influenza viral ribonucleoprotein (vRNP) for CRM1-mediated nuclear export, thereby ensuring the efficient export of host antiviral transcripts. The whole picture of this study is summarized in **Figure 7** to indicate that the phosphorylated CASK enters nucleus and forms a complex to facilitate CRM1-mediated IFNA mRNP export during H5N1 infection.

## Discussion

CASK, a multifaceted scaffolding protein, is involved in various cellular processes such as synapse formation, neuronal development, and gene expression regulation. Mutations in the human CASK gene are associated with X-linked brain malformations and mental retardation (2). Despite its abundant expression in myeloid cells, CASK’s role in immune response regulation remains unexplored. Although primarily localized in the cytosol, co-transfection of CASK with Tbr-1, a crucial T-box transcription factor in forebrain development, triggers CASK’s translocation into the nucleus, where it binds to a specific DNA sequence called the T-element to regulate gene expression (9). However, the signals governing CASK’s movement from the membrane to the nucleus, as well as its role in regulating gene expression in immune cells upon activation, have not been investigated.

In this study, we found that H5N1-IAV infection activates PKR, leading to the upregulation of CASK expression and its subsequent translocation to the nucleus. This translocation of CASK is initiated by the activation of the Src family kinase HCK, which induces the phosphorylation of CASK at residue S395 via CDK5. Notably, mutation of CASK at residue S395 abolishes nuclear translocation triggered by HCK, indicating the crucial role of CASK phosphorylation in this process. Based on our data, we hypothesize that the nuclear translocation of CASK in response to H5N1 infection requires two distinct signals: activation of PKR and the HCK-CDK5 axis. PKR is activated by 5’ppp dsRNA, while, according to the literature, the upstream signal for HCK may involve TLR3. TLR3 activation directly induces the phosphorylation of SRC at Y416 (30), leading to its activation. Notably, HCK is a member of the SRC-family kinases (SFK). This two-signal hypothesis explains why TLR3 activation alone is insufficient to drive the nuclear translocation of CASK.

PKR is known to phosphorylate eIF2α, which inhibits the recycling of eIF2β, thereby blocking translation initiation and exerting a global translational inhibition effect. However, studies have shown that under PKR activation, a subset of mRNAs with complex secondary structures and multiple upstream short open reading frames (uORFs) in their 5’ UTR can escape this inhibition. This is because the translation of these mRNAs is regulated at the elongation stage rather than initiation (31). Notably, the mouse Cask mRNA transcript is 8,376 bp long, with a 490 bp 5’ UTR and a 5,106 bp 3’ UTR. Similarly, the human CASK mRNA transcript is 8,749 bp long, featuring a 566 bp 5’ UTR and a 5,487 bp 3’ UTR. These extended 5’ and 3’ UTRs likely play a critical role in regulating the translation of Cask mRNA, enabling CASK expression under PKR activation.

Moreover, CASK regulates the transport of *Ifna* mRNA from the nucleus to the cytosol, with a significant decrease in IFN-α secretion in CASK-deficient macrophages during H5N1 infection. Furthermore, the myeloid-specific knockout of CASK impedes IFN-α secretion and exacerbates pulmonary inflammation following H5N1-IAV infection. Although CASK knockout does not affect viral replication or titer, it significantly impairs the nuclear export of *Ifna* mRNA, leading to a reduction in interferon-alpha (IFN-α) secretion. This reduction disrupts the activation of interferon-stimulated genes (ISGs), which are essential for controlling inflammation and orchestrating antiviral defense. As a result, the absence of CASK creates a cytokine signaling imbalance, allowing unchecked pro-inflammatory responses to dominate. This dysregulation contributes to severe inflammatory phenotypes, including heightened lung inflammation in CASK-deficient mice. Furthermore, the findings suggest that without CASK, competition for CRM1-mediated nuclear export machinery favors viral mRNA, further undermining the host’s ability to mount an effective immune response. These observations highlight the significant role of CASK in host defense against H5N1 IAV infection through the regulation of IFN-α production and reveal a novel mechanism of CASK-mediated regulation of gene expression via facilitating mRNA transportation from the nucleus to the cytosol.

It is interesting to note that CDK5 phosphorylate residue S395 located in the L27 domain of CASK protein. The L27 domain, protein-protein interaction module, can form heteromeric complexes that integrate multiple scaffold proteins into supramolecular assemblies required for the establishment and maintenance of cell polarity (32). The L27 domain can occur singly or in duplication in association with other domains such as PDZ, SH3, the guanylate kinase domain, or the serine/threonine protein kinase domain, as seen in the CASK protein. Interestingly, SAP97, a membrane-associated guanylyl kinase (MAGUK) protein crucial for trafficking various proteins essential to cellular functions, undergoes phosphorylation by CaMKII kinase at S39 located within the N-terminal L27 domain. This phosphorylation event modulates the trafficking of SAP97 and its associated proteins (33). Consequently, phosphorylation of the L27 domain appears to play a pivotal role in regulating protein assembly among members of MAGUK family.

In the nervous system, CDK5 is involved in neuron migration, neurite outgrowth and support, and synaptogenesis. Furthermore, CDK5 phosphorylates the L-type voltage-dependent Ca^++^ channel (L-VDCC), inhibiting the exocytosis of insulin and thereby regulating glucose-stimulated insulin secretion (34). Given CASK’s involvement in brain development and insulin vesicle secretion within pancreatic beta-cells, it would be interesting to ask whether nuclear translocation of CASK is involved in CDK5-mediated neuron migration, synaptogenesis, and insulin secretion. In addition to CDK5, CASK is phosphorylated by protein kinase A (PKA) at residue T724 in the guanylate kinase domain to increase its interaction with Tbr1 (35). Furthermore, it has been reported that CDK5 phosphorylates residue S51, S395, and T846 of CASK in neuronal cells, resulting in CASK distribution to membrane to promote synaptogenesis (28). In this study, we found that H5N1-IAV causes CASK phosphorylation at S395 via the HCK-CDK5 axis, resulting in the nuclear translocation of CASK in macrophages. Interestingly, we did not detect tyrosine phosphorylation of CASK after H5N1 infection. Instead, we found that CASK is phosphorylated at S395, and a CDK5 inhibitor is able to suppress CASK’s nuclear translocation. This observation suggests that HCK-mediated CASK translocation is regulated through CDK5 activation, thus it is interesting to investigate how HCK regulates CDK5 activation in macrophages in the future.

While HCK plays a crucial role in regulating IAV-induced nuclear translocation, the mechanism by which IAV infection activates HCK remains unclear. Currently, there is no conclusive evidence supporting the activation of HCK via PKR- or RIG-I-mediated pathways. However, it has been demonstrated that TLR ligands can activate HCK through a MyD88-dependent mechanism (36). Since IAV is known to activate TLR3 (37), which signals via MyD88 and TRIF, it is plausible that IAV-induced HCK activation occurs through the TLR3-MyD88-HCK pathway. Additionally, the PKR inhibitor is as effective as the CDK inhibitor in suppressing CASK nuclear translocation, raising the intriguing possibility that PKR may directly phosphorylate CASK and interact with the HCK-CDK5 axis. Furthermore, whether the PKR and SRC can activate CASK independently need to be explored in the future.

CASK selectively regulates IFN-alpha but not IFN-beta, suggesting an evolutionary advantage in having these two antiviral type I IFNs governed by distinct regulatory mechanisms. This diversity potentially confers greater benefits in overcoming viral attacks. While there is only one IFN-beta gene, mice possess 14 *Ifna* isotypes and humans have 13. Influenza A virus NS1 protein interacts with NFX1, the mRNA export receptor for bulk mRNA transport, thereby blocking the nuclear export of poly-A tailed mRNAs (38), including IFN-beta. In contrast, *Ifna* mRNA nuclear export is CRM1-mediated (39). The high nucleotide sequence conservation in the coding regions containing cis-active structures (40) suggests that all *Ifna* isotypes likely utilize a CRM1-dependent export pathway. In the context of influenza A virus infection, competition for CRM-1 mediated mRNA export machinery between viral ribonucleoprotein (vRNP, viral genome) (41) and *Ifna* mRNA is intense. Our findings elucidate a CASK-dependent host strategy that counteracts this competition, favoring the nuclear export of *Ifna* mRNA.

In addition to H5N1 influenza A virus, many other RNA and DNA viruses exploit the host’s CRM1-mediated nuclear export pathway for viral mRNA export. These include Human Immunodeficiency Virus 1, Human T-cell leukemia virus type 1, Rous Sarcoma Virus, Prototype foamy virus, and Human papillomavirus (42). Whether CASK has evolved to counteract these viruses’ competition for CRM1-mediated nuclear export and promotes the expression of host interferon-α (IFNA) remains to be investigated.

The model presented in **Figure 7** is based on interactome data obtained through co-immunoprecipitation followed by proteomic analysis. In addition to host proteins, we identified the interaction between the IAV nucleoprotein (NP) with CASK. Previous studies have demonstrated that IAV NP directly interacts with CRM1 (41), suggesting that CASK may associate with IAV NP either via CRM1 or through direct interaction. Further investigation, including independent validation of these proteomics findings through IP experiments, is needed to determine whether CASK can directly interact with viral proteins, primary interactants and secondary interactants. Moreover, the use of a phosphorylation-null CASK mutant (CASK S395A) could clarify whether CASK phosphorylation contributes to IFN-α expression.

Our findings indicate that CASK’s role in *Ifna* mRNA export may extend beyond H5N1 infection, offering a framework to understand similar mechanisms in other viral contexts. These results fill a critical gap in understanding the intracellular processes governing *Ifna* mRNA export in macrophages and its broader implications for immune responses. The described mechanism—CASK-mediated nuclear translocation and CRM1-dependent mRNA export—could be leveraged to address competition between host and viral mRNAs for nuclear export machinery. Future research should investigate CASK’s involvement in regulating immune responses across various viral infections to elucidate its therapeutic potential further.

This study uncovers a novel role for CASK as a key regulator of *Ifna* mRNA export during H5N1 infection. By serving as a hub for protein-protein interactions, CASK facilitates the efficient nuclear export of antiviral transcripts. This function is analogous to that of other scaffold proteins, such as MAVS and NEMO, which coordinate antiviral signaling. Notably, our findings suggest that CASK’s regulatory functions might also apply to other RNA viruses requiring robust interferon responses for viral control. Supporting this hypothesis, our data (not shown) indicate that dengue virus, another single-stranded RNA virus, similarly induces the nuclear translocation of CASK in primary macrophages. These insights not only clarify CASK’s mechanism of action but also underscore its potential as a therapeutic target to mitigate hyperinflammatory responses during severe viral infections.

In addition to *Ifna* mRNA export, CASK may also influence *Ifna* expression at the translational level. This is supported by the observation that cytoplasmic *Ifna* mRNA is more abundant than nuclear *Ifna* mRNA, yet the differences between WT and CASK KO mice remain relatively subtle (**Figure 2**). Considering CASK’s function as a scaffold protein, investigating its cytoplasmic interactome following IAV infection would be a valuable approach to better understand its role in the cytoplasm.

## Methods

### Reagents and antibodies

Culture media and supplements were purchased from Invitrogen GIBCO. Chemical reagents were from Sigma. Granulocyte–macrophage-colony-stimulating factor (GM-CSF) was from R&D systems. PKR inhibitor was purchased from Santa Cruz (sc-204200) and SFK inhibitor (PP2) was purchased from sigma (529573). 5’pppRNA (tlrl-3prna), 5’ppp dsRNA control (tlrl-3prnac), CL097 (tlrl-c97) and polyI:C (tlrl-pic) were purchased from InvivoGen. Antibodies used in western blot, immunostaining and immunoprecipitation are listed as follows (**Table M1**):

**Table M1:** Antibodies for WB, IF, and IP.

Primary antibodies			
Name	Host species	Clone No.	Source
Anti-CASK	mouse	S56A/50	Sigma, SAB5200069 (for IF)
Anti-CASK	mouse	K56A/50	Biolegend, 830601 (for WB)
Anti-CASK	rabbit	polyclonal	Abnova, PAB27198 (for IP)
Anti-HCK	mouse	3D12E10	Santa Cruz, 101428 (for IF)
Anti-CDK5	mouse	J3	Santa Cruz, sc-6247 (for IP)
Anti-GFP	rabbit	polyclonal	Abcam, ab290 (for IP)
Anti-GAPDH	mouse	6C5	Sigma, MAB374 (for WB)
Anti-Histone H3	rabbit	polyclonal	Abcam, ab1791 (for WB)
Secondary antibodies			
Name	Label	Clone No.	Source
Goat anti-mouse IgG	HRP	AP181P	Sigma-alderich, AP181P (for WB)
Goat anti-mouse IgG	Alexa-488	polyclonal	Jackson Immuno Research Labs, 115545146 (for IF)
Mouse anti-Rabbit IgG	HRP	monoclonal	Jackson Immuno Research Labs, 211032171 (for WB)
Donkey anti-mouse IgG	Alexa-647	polyclonal	Jackson Immuno Research Labs, 715605150 (for IF)

### Culture of mouse primary GM-Mϕ

Male mice aged 8-10 weeks were euthanized, and bone marrow cells were harvested from their femurs and tibias. The isolated bone marrow cells were then washed with 1× RBC lysis buffer to eliminate red blood cells. Subsequently, the nucleated cells were precipitated by centrifugation at 430 g for 5 minutes at room temperature. After discarding the supernatant, the cell pellet was resuspended in 5 ml of RPMI culture medium. This medium consisted of RPMI-1640 (Hyclone, GE Healthcare Life Science) supplemented with 10% FBS (Sigma-Aldrich), 1× penicillin-streptomycin (Gibco), 50 mM 2-mercaptoethanol (Sigma-Aldrich), and 10 ng/mL mouse GM-CSF (R&D systems). A total of 5×10^6^ cells were then seeded into a 9 cm non-coated, sterile petri dish. The culture was maintained by replenishing with fresh medium every 2-3 days for a total of 7 days prior to experimental use.

### H5N1 infection of primary GM-Mϕ

Throughout this study, an MOI of 1 was employed for H5N1 infection of GM-MΦ. For RNA isolation and ELISA experiments, GM-MΦ were seeded in 12-well culture plates at a density of 1×10^6^ cells per well. Each well was infected with 0.5 ml of virus suspension (2×10^6^ pfu/mL H5N1) and incubated at 37°C for 1 hour. Subsequently, the virus-containing medium was replaced with 1 ml of fresh culture medium per well. For Western blot experiments, 2×10^7^ GM-MΦ were infected with 4 ml of virus suspension (5×10^6^ pfu/mL H5N1) in 50 ml conical tubes and incubated at 37°C for 1 hour. Following incubation, the virus-containing medium was removed, and the cells were transferred to 9-cm Petri dishes containing 10 ml of RPMI culture medium per dish. H5N1 infected GM-MΦ were then incubated at 37°C for indicated hours post infection.

### Stimulation of RIG-I, PKR and MDA5 through transfection of the PRR ligand 5’pppRNA and poly(I:C) in GM-Mϕ

GM-MΦ (1×10^5^) were seeded overnight on acid-etched coverslips (High Precision Microscope Cover Glasses, 12 mm, No. 1.5H; Paul Marienfeld GmbH & Co. KG, 0117520) placed in 24-well plates with 0.5 mL of medium per well, prior to experimentation. Transfection of 5’pppRNA and poly(I:C) was performed using Lipofectamine 2000 (Invitrogen, 11668027). For the transfection mixture: Combine 50 μL of OPTI-MEM (Gibco, 31985070) with 2 μL of 0.1 μg/μL ligand stock in a 1.5 mL Eppendorf tube labeled “A.” In a separate 1.5 mL Eppendorf tube labeled “B,” mix 50 μL of OPTI-MEM with 2 μL of Lipofectamine 2000. Gently mix the contents of tubes A and B and incubate for 20 minutes at room temperature. The transfection mixture was then added to the GM-MΦ cultures in the 24-well plates containing 400 μL of culture medium per well. Cells were incubated at 37°C in a 5% CO_2_ incubator for 3 or 6 hours, after which they were fixed with 4% paraformaldehyde (Electron Microscopy Sciences, 15710).

### Fluorescent confocal microscopy

GM-MΦ or 293T cells (1×10^5^) were seeded overnight on acid-etched coverslips (High precision microscope cover glasses 12 mm, No. 1.5H (Paul Marienfeild GmbH & co. KG, 0117520)) in 24-well plates, with 0.5 mL medium per well, prior to experimentation. Following treatment, cells were washed and fixed with 300 μl of 4% paraformaldehyde (Electron Microscopy Sciences, 15710) in PBS for 20 minutes, then washed twice with 300 μl of 1× PBS. Cells were permeabilized with 0.5% Triton X-100 in 1× PBS for 10 minutes, followed by two PBS washes. Blocking was performed using 10% BSA in PBS for 1 hour at room temperature, followed by two additional PBS washes. Primary antibody incubation was conducted overnight at 4°C using antibodies diluted 1:50 in 3% BSA/PBS. For CASK staining, anti-CASK antibody (Sigma, SAB5200069) was used, while anti-HCK antibody (Santa Cruz, 101428) was employed for HCK staining. After two PBS washes, cells were incubated with secondary antibodies (diluted 1:200) for 1 hour. Goat anti-mouse Alexa-488 (Jackson ImmunoResearch Labs, 115-545-146) was used for CASK detection, and donkey anti-mouse-Alexa 647 (Jackson ImmunoResearch Labs, 715-605-150) for HCK detection. Following secondary antibody staining, cells were washed twice with 1× PBS and incubated with 0.1 μg/mL Hoechst 33342 in 1× PBS for 30 minutes at room temperature. After two final PBS washes, slides were prepared for imaging. Image acquisition was performed using a Leica TCS SP8 X-FALCON confocal microscope. To determine optimal fluorescence gain settings, secondary-antibody-only controls were employed, which should exhibit minimal signal under the selected gain values.

### Influenza A virus H5N1 propagation and titer determination by plaque assay

H5N1 in this study is derived from recombinant PR8 strain expressing HA and NA from VN1203 (VN HA, NA) and was generated as described previously (43). The multi-basic HA cleavage site within VN HA, NA strain is replaced by that of a low-pathogenicity avian influenza virus (44). The H5N1 virus is cultured and expanded using MDCK cells. Virus titer is determined using MDCK cells for plaque assay (45).

### Cytokine measurement by ELISA and multiplex immunoassay

The concentrations of mouse IFN-α in media from macrophages were measured by ELISA according to the manufacturer’s instructions (Invitrogen, BMS6027).

The level of mouse IL-1β, IFN-β, IL-6, TNF-α, IP-10 and IFN-γ in GM-Mϕ culture supernatant was measured by Multi-Plex Immunoassay (MPI) performed by Inflammation Core Facility (Institute of Biomedical Sciences, Academia Sinica, Taiwan). Antibody conjugated magnetic beads were incubated with cytokine containing samples, washed and incubated with biotinylated antibody and subsequently Streptavidin-Phycoerythrin (PE). The fluorescence levels of the beads were measured by Bio-Plex^®^ 200 system (Bio-Rad, USA) and the concentration of the cytokines were calculated with standard. All assays were protected from light and performed at room temperature.

### Extraction of total cell lysate for western blot and ELISA

Prepare a cell lysis solution containing 50 mM Tris-HCl (pH 7.4), 150 mM NaCl, 1% Triton X-100, 1X Phosphatase Inhibitor Cocktail (Roche, 04906837001), and 1X Protease Inhibitor Cocktail (Roche, 04693132001). Resuspend the cell pellet in the lysis solution and incubate on a rotary tube mixer at 4°C for 1 hour. Centrifuge the lysed cells at 16,000 g for 10 minutes at 4°C. Collect the supernatant (total cell lysate) into a clean, labeled Eppendorf tube. Dilute the total cell lysate 10X or 20X, and measure the protein concentration using the DC Protein Assay Kit (Bio-Rad, 5000111). For western blot, adjust the samples to equal protein concentrations and mix with 6X sample buffer. Load 25 μl of the samples and separate them by SDS-PAGE using a constant voltage of 50–100V. For IFNA ELISA, the total cell lysate was subjected to buffer exchange using Pierce Protein Concentrators PES, 10K MWCO (Thermo Scientific, 88513) spin columns. The proteins were then resuspended in 120 μl of 1X PBS for the following ELISA experiments.

### Extraction of nuclear proteins for western blot

Nuclear and cytoplasmic fractions were separated using the NE-PER Nuclear and Cytoplasmic Extraction Reagents (Thermo, 78833). To enhance protein preservation, phosphatase inhibitor cocktail (Roche, 04906837001) and protease inhibitor cocktail (Roche, 04693132001) were added to the extraction reagents, with one-quarter tablet of each per 1 mL of CERI reagent (Thermo, 78833) and 0.5 mL of NER reagent (Thermo, 78833).

For each treatment, 2×10^7^ cells were utilized. Cells were washed with 1× PBS and transferred to a 1.5 mL microcentrifuge tube, then pelleted by centrifugation at 500 g for 3 minutes. The supernatant was carefully removed, leaving the cell pellet as dry as possible. The cell pellet was resuspended in 200 μL of ice-cold CERI and incubated on ice for 10 minutes. Subsequently, 11 μL of ice-cold CERII (Thermo, 78833) was added, and the mixture was homogenized by pipetting. After 1 minute of incubation on ice, the tube was centrifuged at 16,000 g for 5 minutes. The supernatant (cytoplasmic extract) was transferred to a clean microcentrifuge tube. To minimize cytosolic contamination, the insoluble pellet was washed with 1 mL of 1× PBS, and the supernatant was carefully removed, leaving the pellet as dry as possible.

The insoluble pellet containing nuclei was then suspended in 100 μL of ice-cold NER. The suspension was vortexed at the highest setting for 15 seconds every 10 minutes, for a total of 5 cycles. Following this, the tube was centrifuged at 16,000 g for 10 minutes. The resulting supernatant (nuclear extract) was transferred to a clean microcentrifuge tube. Protein concentrations in each fraction were determined using the DC™ Protein Assay Kit.

### RNA isolation, reverse transcription and qPCR

Monolayer cells in 12-well (1×10^6^ cells) are lysed with 0.4 ml Trizol^®^Reagent (Invitrogen, 15596-026), homogenizing the lysate by vortex for 1 minute. Using TriRNA Pure Kit (Geneaid, TRP200) for RNA extraction and following the manufacture’s instruction. RNA concentrations were measured using NanoDrop Microvolume Spectrophotometers.

Reverse transcription was performed using RevertAid™ First Strand cDNA Synthesis Kit (Thermo, K1622). 1 µg of RNA was dissolve in RNase-free water in a final volume of 23 µl followed by adding 2 µl of 100 µM oligo(dT) primer and 1 µl of RiboLock™ Ribonuclease Inhibitor (20U/µl) (Thermo Scientific, EO0381), then incubate at 65°C for 5 minutes. Placing the tube on ice and add the following components: 5X reaction buffer 8 µl, 10 mM dNTP mix 4 µl, and Reverse transcriptase 2 µl. Mix gently then incubate the mixture at 42°C for 60 minutes. Terminating the reaction by heating at 72°C for 10 minutes, and store cDNA samples in -20°C.

40 µl cDNA samples are diluted to 200 µl for the following real-time PCR reaction. Using 2 µl diluted cDNA, 5 µl 2× Luminars Color HiGreen qPCR Master Mix (Thermo Scientific, K0393), 2.4 µl d_2_H_2_O, 0.6 µl 10 µM (forward primer + reverse primer) for each reaction in 384 well. The reaction condition is as follows: hot start 95°C, 5 minutes, denaturing 95°C, 10 seconds, annealing 60°C, 1 minute, elongation 72°C, 10 seconds, 40 cycles from denaturing to elongation. Primers for qPCR are listed as follows (**Tables M2** and **M3**):

**Table M2:** qPCR primers for *Cask, Gapdh*, cytokines and chemokines.

species	control	f/r	primer sequence
mouse	Cask	forward	CTATGCGAGGTGATCGGCAA
	Cask	reverse	GCTTCCCGCTTTAGATCTTCTGT
mouse	Gapdh	forward	GGGTCCCAGCTTAGGTTCAT
	Gapdh	reverse	CCAATACGGCCAAATCCGTTC
species	cytokines	f/r	primer sequence
mouse	Tnf-a	forward	TAGCCCACGTCGTAGCAAAC
	Tnf-a	reverse	TGTCTTTGAGATCCATGCCGT
mouse	Il-6	forward	AGTTCCTCTCTGCAAGAGACTTC
	Il-6	reverse	TCTCCTCTCCGGACTTGTGAA
mouse	Ifnb1	forward	AGTACAACAGCTACGCCTGG
	Ifnb1	reverse	GAGGCATCAACTGACAGGTCT
mouse	Ifnb1	forward	CAACCTCACCTACAGGGCG
	Ifnb1	reverse	TGGATGGCAAAGGCAGTGTA
mouse	Ifna4	forward	GCCTTGACAGTCCTGGAAGAA
	Ifna4	reverse	GAGCCTTCTGGATCTGTTGGT
mouse	Ifng	forward	TCCAGCGCCAAGCATTCAA
	Ifng	reverse	GGGACAATCTCTTCCCCACC
mouse	IfnL2	forward	GAAGGTCTGGGAGAACATGACTG
	IfnL2	reverse	CTGGGAGTGAATGTGGCTCAG
species	Chemokines	f/r	primer sequence
mouse	Ip-10 (Cxcl10)	forward	GCTGAGAGACATCCCGAGC
	Ip-10 (Cxcl10)	reverse	CTTGAGTCCCACTCAGACCC
mouse	Mcp-1(Ccl2)	forward	TCACCAGCAAGATGATCCCAA
	Mcp-1(Ccl2)	reverse	CTTGAGCTTGGTGACAAAAAC TACA
mouse	Mip-1B (Ccl4)	forward	CCCAGCTCTGTGCAAACCTA
	Mip-1B (Ccl4)	reverse	CCATTGGTGCTGAGAACCCT

**Table M3:** qPCR primers for Interferon-stimulated genes (ISG) and (Src-family kinases (SFK).

species	ISG (Interferon-stimulated gene)	f/r	primer sequence
mouse	Stat1	forward	GTCATCCCGCAGAGAGAACG
	Stat1	reverse	GCAGAGCTGAAACGACCTAGA
mouse	Il-1B	forward	GCCACCTTTTGACAGTGATGAG
	Il-1B	reverse	ATGTGCTGCTGCGAGATTTG
mouse	Il-18	forward	CAAGTTTACAAGCATCCAGGC ACAG
	Il-18	reverse	GATTTGGAAGGTTTGAGGCGG
mouse	Nlrp3	forward	TCGTCACCATGGGTTCTGGTC
	Nlrp3	reverse	TCCTGAGCCATGGAAGAAAAGT
mouse	Irf7	forward	AGCTTGGATCTACTGTGGGC
	Irf7	reverse	CCCGGCATCACTAGAAAGCA
mouse	Tlr3	forward	CGGCCTAGTTGTTTCTGGGA
	Tlr3	reverse	CAGCCTGAAAGTGAAACTCGC
mouse	Tlr7	forward	AAGAAAGATGTCCTTGGCT CCC
	Tlr7	reverse	ATGTCTCTTGCTGCCCCAAA
mouse	Rig-i	forward	GGCTGAAAGCAAGGCTGATG
	Rig-i	reverse	AAGACGCTTCTGAAGGAGGC
mouse	Mavs	forward	CTTGGCTAGGGCCAGTTACG
	Mavs	reverse	TGGACTGAGATGGACTGCTT
mouse	Sting	forward	CCGATTTCCGGGGGATCAAT
	Sting	reverse	TCTGAATGGGGAGACAGCAG
mouse	Pkr	forward	CCGGGAAAACGAAACAGAA GAG
	Pkr	reverse	TCCCAGTGGCCAAAGTTTCTG
mouse	Mda5	forward	CCCAGAAGACAACACAGACTTG
	Mda5	reverse	GCCTCTGTCTCCAGACTTGAC
species	Sfk (Src family kinase)	f/r	primer sequence
mouse	Csk	forward	GGCCTGGACCGCAGTG
	Csk	reverse	AGGCGGCCTGTATTGCC
mouse	Fyn	forward	GACCATGTGAATGTGCTCCG
	Fyn	reverse	CGGACTGACTTTTTGCCACG
mouse	Hck	forward	TTCTTCAAGGGGATCAGCCG
	Hck	reverse	CGAGTAGCTCCCTTTGGTGG
mouse	Fgr	forward	GCAGAGGCGGGTAGCAC
	Fgr	reverse	GAGGTGCCGGAAACCTGG

### Myeloid-specific CASK knockout mice: generation, breeding strategy and genotyping

#### Generation of CASK flox mice by CRISPR-mediated gene editing

Cask^flox^ mice were generated by CRISPR-mediated pronuclei genome editing. Lox P sites are designed to be flanking CASK exon 2 following a stepwise procedure. Lox P site was first inserted into 5 prime side of CASK exon 2 in pronuclei and followed by *in vitro* fertilization and zygote microinjection into surrogate mother mice. The littermates are screened for 5 prime lox P site by genotyping. Pronuclei from the oocytes of female carriers are used for 3 prime lox P site insertion and *in vitro* fertilization. The littermates are screened for 5 prime lox P site and 3 prime lox P site. Double positive male carriers are founders of CASK^flox^ strain.

Cask^flox^ mice are crossed with C57BL/6JNarl mice (provided by National Laboratory Animal Center (NLAC), NARLabs, Taiwan) for 3 generations to dilute and minimize potential CRISPR-mediated off-target effects. N4 Cask^flox^ mice are then crossed with LysM-Cre mice to generate myeloid-specific Cask knockout offsprings. Male offsprings with genotype Cask^flox^ LysMCre+ are myeloid-specific Cask KO and Cask^wt^ LysMCre+ are littermate controls.

For genotyping, mouse genomic DNA is obtained from 3 mm tail-tip cut overnight digested using 200 µl Tail Digestion Buffer (0.1M Tris-HCl, 5mM EDTA, 0.5% SDS, 0.2M NaCl) with 0.1 mg/ml Proteinase K (Thermo Scientific, EO0491). After overnight digestion, add 200 µl isopropanol to precipitate gDNA. Centrifuge at 16000 g for 10 minutes at room temperature. Remove and discard supernatant. Air dry the pellet then dissolve gDNA in 60 µl autoclaved d_2_H_2_O. gDNA is then used for genotyping PCR. The PCR condition is as follows: Initial denaturation 95°C 1 minute, denaturation 95°C 20 seconds, annealing 58°C, 20 seconds, elongation 72°C, 1 minute, final extension 72°C 7 minutes, 50 cycles from denaturation to elongation. Each mouse was genotyped for 5 prime loxP site, 3 prime loxP site, LysMCre mutant and LysMcre wildtype. Genotyping primers are listed as follows (**Tables M4**):

**Table M4:** Primers for CASK^flox^ and LysMCre.

Target: CASK	Primer Name	sequence 5’ to 3’	PCR Product size
5’ loxp site	Cask 5VF4	GAATGGGTCTGGGGGCTTC	WT: 507 bp
	Cask 5VR4	CGGCTCTACCACGCTAACCA	Target: 547 bp
3’ loxp site	Cask 3VF1	GGGCAAAGGCCAGATACATG	WT: 239 bp
	Cask 3VR1	TCCCCAGCCCTTCATTTGTC	Target: 279 bp
Exon 2 KO	Cask 5VF4	GAATGGGTCTGGGGGCTTC	WT: 78 kb
	Cask 3VR1	TCCCCAGCCCTTCATTTGTC	Target: 643 bp
Target: LysMcre	Primer Name	sequence 5’ to 3’	PCR Product size
LysMCre	oIMR3066 Mutant	CCC AGA AAT GCC AGA TTA CG	Target: 700 bp
(Mutant)	oIMR3067 common	CTT GGG CTG CCA GAA TTT CTC	
LysMCre	oIMR3068 WT	TTA CAG TCG GCC AGG CTG AC	WT: 350 bp
(WT)	oIMR3067 common	CTT GGG CTG CCA GAA TTT CTC	

C57BL/6J mice were purchased from the National Laboratory Animal Center (NLAC, Taiwan). All mice were bred and maintained in specific pathogen-free conditions at Academia Sinica SPF animal facility (AS core). Animal experiments were approved by the Institutional Animal Care and Use Committee (IACUC) at AS core (protocol ID 19-02-1288). In this study, we used 8–12-week-old male.

### Interferon alpha mRNA subcellular localization measured by RNA FISH and fluorescence confocal microscopy

The probe sequences are designed using online software (Stellaris Probe Designer version 4.2) (46). Specific probes, 24 probes each, for Ifna2 and Ifna4 are combined to represent Interferon alpha mRNA, and ordered as Stellaris RNA FISH probes, custom assay with Quasar 570 dye. Probes are reconstituted to 12.5 µM using TE buffer (10 mM Tris-HCl, 1mM EDTA, pH8.0). Probe sequences are listed as follows (**Tables M5**):

**Table M5:** Mouse Ifna RNA FISH probe sequences.

Mouse Ifna4 Probe sequences	Mouse Ifna2 Probe sequences
1.tctgggctgtgggtttga	1. agcacagagtctagccat
2. gggtcttgtagatgctgg	2. ctatcagcatcacgagga
3. cacagagcctagccattg	3. ttgaccagtagctcatca
4. ttaccaggatcatgagga	4. cgcatcctagagaacagg
5. accagtagtagctcatca	5. ggttataagtgtgaggca
6. catcctagagaacaggct	6. ttcaaggccctcttgttc
7. taagtgtgaggcaggtca	7. ctcatctgtgccaggacc
8. ctcttgttcccgaggtta	8. aagtcctgcctgtccttc
9. ttccaggactgtcaaggc	9. ttctccagggggaatcca
10. gggggagtcttctcattt	10. atctgctggttatccacc
11. ccttcaggcaggaaagag	11. atggcttgagccttctgg
12. atccaaaatccttcctgt	12. agatctcgcagcacaggg
13. atccaccttctccaaggg	13. ttcaaggtctgctgagta
14. caaggatggcttgagcct	14. gcctttgatgtgaagagg
15. ctgctgggtaagatctct	15. attccaagcagcagatga
16. gttgcattccaagtagca	16. tgagtctaggagggttgt
17. gctgatggaggtcattgc	17. ggtggaggtcattgcaga
18. gctttgaggtcattgagc	18. tgcaggtcattgagctgc
19. gaggttcctgcatcacac	19. acctgctgcatcagacag
20. agtcttcctgggtcagag	20. ctgggtcagaggaggttc
21. aagtatgtcctcacagcc	21. tatttcctcacagccagc
22. ggtacacagtgatcctgt	22. ggtacacagtgatcctgt
23. ttctgctctgatcacctc	23. gacttctgctctgaccac
24. aagagagggctctccaga	24. aggaagacagggctctcc

Hybridization mixture contains 125 nM probe diluted using Stellaris RNA FISH Hyrbidizatiob Buffer (Biosearch, SMF-HB1-10). Mouse primary GM macrophage were seeded onto coverslip at the density of 6X10^4^/well in 24-well, and subjected to H5N1 infection for indicated time points before fixation using paraformaldehyde (4% paraformaldehyde (Electron Microscopy Sciences, 15710)) in 1XPBS (RNase free) for 15 minutes. The fixed cells are then washed with 1XPBS (RNase free) and immersed in 70% ethanol 1XPBS (RNase free) to make plasma membrane permeable. Use 0.5 ml wash buffer A (20% Wash Buffer A stock, SMF-WA1-60, 10% deionized formamide in ultrapure nuclease-free water) to immerse the coverslips (High precision microscope cover glasses 12 mm, No. 1.5H (Paul Marienfeild GmbH & co. KG, 0117520)) for 5 minutes before hybridization. For hybridization step, 100 µl of hybridization mixture were used to immerse the coverslip and incubate at 37°C for 4 hours in dark. Followed by wash step 1, use 0.5 ml wash buffer A (20% wash buffer A, SMF-WA1-60, 10% deionized formamide in ultrapure nuclease-free water) to immerse the coverslips at 37°C for 30 minutes in dark. Aspire the wash buffer and stain the fixed cells with DAPI using 0.5 ml DAPI nuclear stain (0.5 ng/ml DAPI in Wash buffer A) at 37°C for 30 minutes in dark. Aspire DAPI nuclear stain and immerse the coverslip with wash buffer B (20% Wash Buffer B stock, SMF-WB1-20) at room temperature for 5 minutes in dark. Dry the coverslips by dripping off wash buffer B and mount the coverslip onto microscope slide using anti-fade mounting solution. Seal the coverslip with nail polish and allow to dry before storing at -20°C. Use confocal microscope to capture 570 nm emission light and record interferon alpha mRNA subcellular localization. RNA FISH confocal images were analyzed for nucleus/cytoplasmic ratio using MetaMorph software.

### CASK EGFP fusion protein and constitutively active HCK constructs

CASK coding sequence were constructed into pEGFP-N vector (Addgene, 6085-1) in EcoRI site to make N-terminal EGFP fusion CASK. Constitutively active HCK(Y499F) were constructed into pCDNA4-V5/His-A vector (Addgene, V86120) in EcoRI site by site-directed mutagenesis.

### Co-overexpression of CASK-EGFP fusion protein and HCK^CA^ in 293T

15 µg of CASK EGFP fusion protein vector and 15 µg of constitutively active HCK Y499F (HCK^CA^) were mixed in 500 µl Opti-MEM (Gibco, 31985070) with 30 µl of Lipofectamine 2000 (Invitrogen, 11668027) to form complex for transfection of each 10-cm culture dish (1× 10^7^) 293T cells for 24 hours.

### Identification of HCK-induced CASK phosphorylation site by IP-LC-MS/MS

10 × 10^7^ 293T cells were transfected with CASK-EGFP and HCK^CA^ for 24 hours to yield 10 mg of protein for immunoprecipitation. Lysate was precleared by 40 µl of 50% Protein A beads (rProtein A Sepharose Fast Flow (GE Healthcare, 17-1279-02)) in final 1 ml wash buffer (50 mM Tris-HCl, pH 7.4, 150 mM NaCl, 0.05% Triton-X100, 1X Phosphatase inhibitor cocktail (Roche, 04906837001), 1X Protease inhibitor cocktail (Roche, 04693132001)), and on rotary tube mixer at 4°C for 1 hour.

To pull down CASK-EGFP fusion protein, we use anti-GFP protein A beads (rProtein A Sepharose Fast Flow (GE Healthcare, 17-1279-02)). Preparation of Anti-GFP Protein A Beads: Mix 40 µl of 50% protein A with 5 µl anti-GFP (Abcam, ab290) in final 1 ml binding buffer. The binding buffer contains 50 mM Tris-HCl, 150 mM NaCl, 0.1% Triton-X100, 1 pill of phosphatase inhibitor cocktail (Roche, 04906837001) and 1 pill of protease inhibitor cocktail (Roche, 04693132001). Incubate the mixture on a rotary tube mixer at 4°C for 2 hours. Wash with 1 ml wash buffer for 3 times by microcentrifuge at 1600g for 1 minute.

Precleared lysate (10 mg) and anti-GFP Sepharose beads were incubated in a final volume of 1 mL at 4°C for 2 hours on a rotary tube mixer. The beads were washed three times with 1 mL wash buffer, once with 1 mL wash buffer containing 0.1% SDS, and once with 1 mL PBS. 50 μL of 2× SDS sample buffer was added to the post-IP protein A Sepharose beads, which were then boiled at 95°C for 10 minutes before SDS-PAGE. SDS-PAGE was performed using a 10% SDS-acrylamide gel, and the gel was stained with Rapid stain (786-31, G-BIOSCIENCES) to visualize the CASK-EGFP band. The gel band corresponding to the CASK-EGFP molecular weight (130 kDa) was excised. In-gel digestion of CASK-EGFP was performed using trypsin (Thermo Scientific, 90059) and chymotrypsin (Thermo Scientific, 90056). The resulting peptides were extracted and subjected to LC-MS/MS for screening of CASK phosphorylation modifications. The mass spectrometry proteomics data have been deposited to the ProteomeXchange Consortium via the PRIDE partner repository with the dataset identifier PXD057889 and 10.6019/PXD057889 for “CASK phosphorylation pattern in the presence of constitutively active HCK”.

### Prediction of phosphorylation sites and its corresponding upstream kinases by NetPhos-3.1

We submit full length CASK 926 amino acids sequence (NP_001271432.1) and CDK5 292 amino acids sequence (NP_031694.1) in FASTA format, respectively, for NetPhos-3.1 (DTU Health Tech) prediction. Our IP-LC-MS/MS study shows that CASK S935 is upregulated upon co-transfection with constitutively active HCK. We then use NetPhos3.1 to predicts which kinase among the 17 kinases (ATM, CKI, CKII, CaM-II, DNAPK, EGFR, GSK3, INSR, PKA, PKB, PKC, PKG, RSK, SRC, cdc2, cdk5 and p38MAPK.) can phosphorylate CASK at S395. CDK5 has a score above 0.5 and hence the positive prediction. We then ask which tyrosine kinase is upstream to CDK5. NetPhos3.1 shows that SRC has a score above 0.5 (0.591), a positive prediction kinase on CDK Y15 site.

### Intranasal challenge of H5N1 LD50 (750 pfu) to CASK^flox^LysMCre and CASK^wt^LysMCre mice, lung lysate preparation for IFNA ELISA and lung histopathology

8-12-week old *Cask* ^flox^LysMCre^+^ and *Cask* ^wt^LysMCre+ mice were anesthetized by intraperitoneal. injection of 150 mg/kg ketamine (Imalgene 1000, Boehringer Ingelheim) and 30 mg/kg Xylazine (Rompun, Dechra). Followed by intranasal inoculation with H5N1 at LD50 (750 pfu in 20 ul serum-free DMEM/mouse) through left nostril. Mice body weights were recorded daily and mice were sacrificed by isoflurane anesthesia followed by cervical dislocation on days 3 and 5 post-infection. The left lungs were collected and homogenized in 1 ml of 1 X PBS supplemented with protease inhibitors (Roche, REF04693132001) and phosphatase inhibitor (Roche, REF04693132001) using MagNA Lyser (Roche) and MagNA Lyser Green Beads (Roche) at 6500 speed, 40 seconds twice with a 10 minutes interval on ice. Lung homogenates were then centrifuged at 160000g for 10 minutes, 4°C, and supernatant were collected and store at -80°C overnight, and were used in IFNA ELISA (Mouse IFN-α (Invitrogen, BMS6027) right next day to prevent loss of IFNA in the homogenates.

For histopathology analysis, the left lungs were fixed in 10% formalin for at least 3 days and then sent to Taiwan Mouse Clinic for paraffin embedding, tissue sectioning and H&E staining. Lung tissue section H&E slides were scanned into “.svs” files by the Leica Aperio GT450. We use the histological scoring system for inflammation in the lungs of mice published by H. K. Bayes et al. (47). Briefly, there are two parts of the scoring system, part one is peribronchial infiltration scoring 0-4, the other part is alveolar involvement scoring 0-3. For peribronchial infiltration, 0=none, 1=mild (infiltrate ≤ 4 cells thick), 2=moderate (infiltrate 5-10 cells thick), 3=severe (infiltrate 25-50% visualized lumens), 4=diffuse (infiltrate > 50% visualized lumens). For alveolar involvement, 0=none, 1=mild (patchy increased cellularity/thickening), 2=moderate (25-50% visualized lung with increased cellularity/thickening), 3=severe (>50% visualized lung with increased cellularity/thickening). We use 4X magnification for histological scoring of left lung cross section. Each cross section is dividing into 15 fields for scoring and then averaged to represent the final score.

### Identification of CASK interactome in resting and activated GM-macrophage by IP-LC-MS/MS

Dynabeads Antibody Coupling Kit (Thermo Fisher, 14311D) was used to covalently couple anti-CASK polyclonal antibody to magnetic beads for immunoprecipitation. Control antibody is Mouse IgG1 (Sigma, M7894), CASK polyclonal antibody (Abnova, PAB27198). Covalent conjugation protocol follows the instruction of Dynabeads Antibody Coupling Kit. Briefly, 10 mg of dynabeads were conjugated with 100 µg of antibody. Activated conjugated beads were used for immunoprecipitation assay. Equal volume (bring volume to 1ml by adding 1% Triton X100 lysis buffer) of nuclear extract from the same treatment were incubated with anti-IgG1 dynabeads (2mg) or anti-CASK dynabeads (2mg). The extract and dynabeads mixture were kept in resuspending using a rotary mixer in 4°C overnight (around 20 hours). The dynabeads were then washed 3 times, 2-minute wash each time, once with 1 ml 0.05% Triton X100 (50 mM Tris-HCl pH7.4, 150 mM NaCl, 0.05% Triton X100, phosphatase inhibitor (Roche, 04906837001) 1 pill, protease inhibitor cocktail (Roche, 04639132001) 1 pill in final 4ml), twice with wash buffer (50 mM Tris-HCl pH7.4, 150 mM NaCl, phosphatase inhibitor (Roche, 04906837001) 1 pill, protease inhibitor cocktail (Roche, 04639132001) 1 pill in final 4ml). Put eppendorf upside down to air dry all remaining liquids before elution. For elution, use 33 µl 0.1 M Glycine pH=3 to immerse the dynabeads and incubate at 55°C for 10 mins. Collect 30 µl of supernatant from each sample and bring the pH to neutral by adding 1.5 µl 1 M ammonium bicarbonate. Store the neutralized sample at -80°C before subjected to LC-MS-MS experiment. The mass spectrometry proteomics data have been deposited to the ProteomeXchange Consortium via the PRIDE partner repository with the dataset identifier PXD058016 and 10.6019/PXD058016 for “Identification of CASK nuclear interactome in resting and H5N1-activated GM-macrophage by IP-LC-MS/MS”.

### LC-MS/MS method

Samples were analyzed by LC-ESI-MS using an Orbitrap Fusion mass spectrometer (Thermo Fisher Scientific, San Jose, CA) equipped with an EASY-nLC 1200 system and EASY-spray source (both from Thermo, San Jose, CA, US). The digestion solution (5 μL) was injected at a flow rate of 1 μL/min onto an EASY column (C18, 0.075 mm × 150 mm, ID 3 μm; Thermo Scientific). Chromatographic separation was performed using 0.1% formic acid in water as mobile phase A and 0.1% formic acid in 80% acetonitrile as mobile phase B, with a flow rate of 300 nL/min. The gradient employed ranged from 5% buffer B at 2 min to 40% buffer B at 50 min.

Full-scan MS conditions were as follows: mass range m/z 375-1500 (AGC target 5E5) with lock mass, resolution 60,000 at m/z 200, and maximum injection time of 50 ms. MS/MS was run in top speed mode with 3 s cycles, using CID for protein identification or HCD and EThcD for phosphopeptide analysis. Dynamic exclusion duration was set to 60 s with a 10 ppm tolerance around the selected precursor and its isotopes. The electrospray voltage was maintained at 1.8 kV, and the capillary temperature was set at 275°C.

### siRNA knockdown of CASK primary interactant *Stip1* and *Cct4*

GM-MΦ 7x10^5^ cells (in 12-well per well) were transfected with (25 µM) siRNAs targeting *Cct4* and *Stip1* for 48 hours prior to H5N1 infection (MOI=1) for 12 hours. Transfection is performed using HiPerFect transfection reagent (Cat. No. 301704, QIAGEN) 12 µL + 5 µL 5µM SMARTpool siRNA in 200 µL Opti-MEM (Cat. No. 31985070, ThermoFisher Scientific) incubate for 10 minutes, and then add drop by drop into 12-well (200 µL RPMI culture medium) to reach final volume of 400 µL. Incubate at 37°C for 4 hours, then add 600 µL RPMI culture medium to reach final volume of 1 mL. *Stip1* siRNA (L-048388-01-0005, ON-TARGETplus SMARTpool, Dharmacon) and *Cct4* siRNA (L-049104-01-0005, ON-TARGETplus SMARTpool, Dharmacon) sequences are listed as follows (**Tables M6**):

**Table M6:** siRNA target sequences.

ON-TARGETplus SMARTpool siRNA	Target sequences
D-001810-10-05, Non-Targeting pool	UGGUU UACAU GUCGA CUAA
D-001810-10-05, Non-Targeting pool	UGGUU UACAU GUUGU GUGA
D-001810-10-05, Non-Targeting pool	UGGUU UACAU GUUUU CUGA
D-001810-10-05, Non-Targeting pool	UGGUU UACAU GUUUU CCUA
J-048388-09, Mouse Stip1	CGAGA GGACU ACCGG CAGA
J-048388-10, Mouse Stip1	CGUCA GACCU GGGCA CGAA
J-048388-11, Mouse Stip1	CCAAU AAUCU CCAGC UUAA
J-048388-12, Mouse Stip1	GGGCA AGGGU UAUUC AAGA
J-049104-09, Mouse Cct4	UGUGG UAAAU ACUCG AUAA
J-049104-10, Mouse Cct4	CAGAU CCGCU UCAGC AAUA
J-049104-11, Mouse Cct4	CGACA GAGAA ACUUU GUUA
J-049104-12, Mouse Cct4	CGUUG AAAGA GAAGA CAUU

Knockdown efficiencies were confirmed by RT-qPCR analysis of *Cct4* and *Stip1* mRNA levels in total RNA extracts. qPCR primer sequences for Stip1 and Cct4 are listed as follows (**Tables M7**):

**Table M7:** qPCR primers for *Stip1* and *Cct4*.

species	CASK primary interactants	f/r	qPCR primer sequence
mouse	*Stip1*	forward	CGAATGCTTCCAGAAAGGGGA
	*Stip1*	reverse	CAGTTTGGCATCTCTCGGGT
mouse	*Cct4*	forward	ATGCTGGCCTGAATCCCATTT
	*Cct4*	reverse	GTTGGAGATCCCACCCTTTCG

### Quantification and statistical analysis

Statistical analyses were performed using GraphPad Prism software (version 9.0; GraphPad Software Inc., San Diego, CA, USA). Data are presented as mean ± standard deviation (SD). Statistical significance between two datasets was determined using either an unpaired, two-tailed Student’s t-test for parametric data or a Mann-Whitney test for nonparametric data. For multiple group comparisons, parametric data were analyzed using one-way ANOVA followed by the Bonferroni *post hoc* test, while nonparametric data were analyzed using the Kruskal–Wallis test followed by Dunn’s *post hoc* test. Across all experiments, significance levels were denoted as follows: *p < 0.05, **p < 0.01, ***p < 0.001, and ****p < 0.0001.

## Data Availability

The original contributions presented in the study are included in the article/**Supplementary Material**. Further inquiries can be directed to the corresponding author.

## Supplementary material

Supplementary Table 1 Source data-confocal images.

Supplementary Table 2 Nuclear CASK interactome raw data.
