# Supplementary material for: Regulation of interferon alpha production by the MAGUK-family protein CASK under H5N1 infection
Source: Front Immunol. 2025 Jan 9;15:1513713. doi: 10.3389/fimmu.2024.1513713 (PMC11754051; doi:10.3389/fimmu.2024.1513713)
Supplement: Supplementary Table 1 — Source data-confocal images. [file Table1.docx]

**Regulation of interferon alpha production**

**by the MAGUK-family protein CASK under H5N1 infection**

**Jing-Ying Huang^1,2^, Pei-Shan Sung^2^, Shie-Liang Hsieh^2,3,4,5*^**

*1Doctoral Degree Program of Translational Medicine, National Yang Ming Chiao Tung University and Academia Sinica, Taiwan*

*2Immunology Research Center, National Health Research Institute, Zhunan, Miaoli, Taiwan*

*3Institute of Clinical Medicine & Institute of Microbiology and Immunology, National Yang Ming Chiao Tung University, Taipei, Taiwan*

*4Department of Medical Research, Taipei Veterans General Hospital, Taipei, Taiwan*

*5Master Program in Clinical Genomics and Proteomics, School of Pharmacy, Taipei Medical University, Taipei, Taiwan*

* **Correspondence:**

Shie-Liang Hsieh ,

[slhsieh@nhri.edu.tw](mailto:slhsieh@nhri.edu.tw)

Link to raw data: <https://drive.google.com/drive/folders/1oRgIFNP4O6IV69KwIhIR2_akOjKtUCG0?usp=sharing>
