# Supplementary material for: Regulation of interferon alpha production by the MAGUK-family protein CASK under H5N1 infection
Source: Front Immunol. 2025 Jan 9;15:1513713. doi: 10.3389/fimmu.2024.1513713 (PMC11754051; doi:10.3389/fimmu.2024.1513713)
Supplement: Supplementary file 4 [file DataSheet1.pdf]

Regulation of interferon alpha production by MAGUK-family protein CASK under H5N1 infection

Supplementary figures

Figure S1.

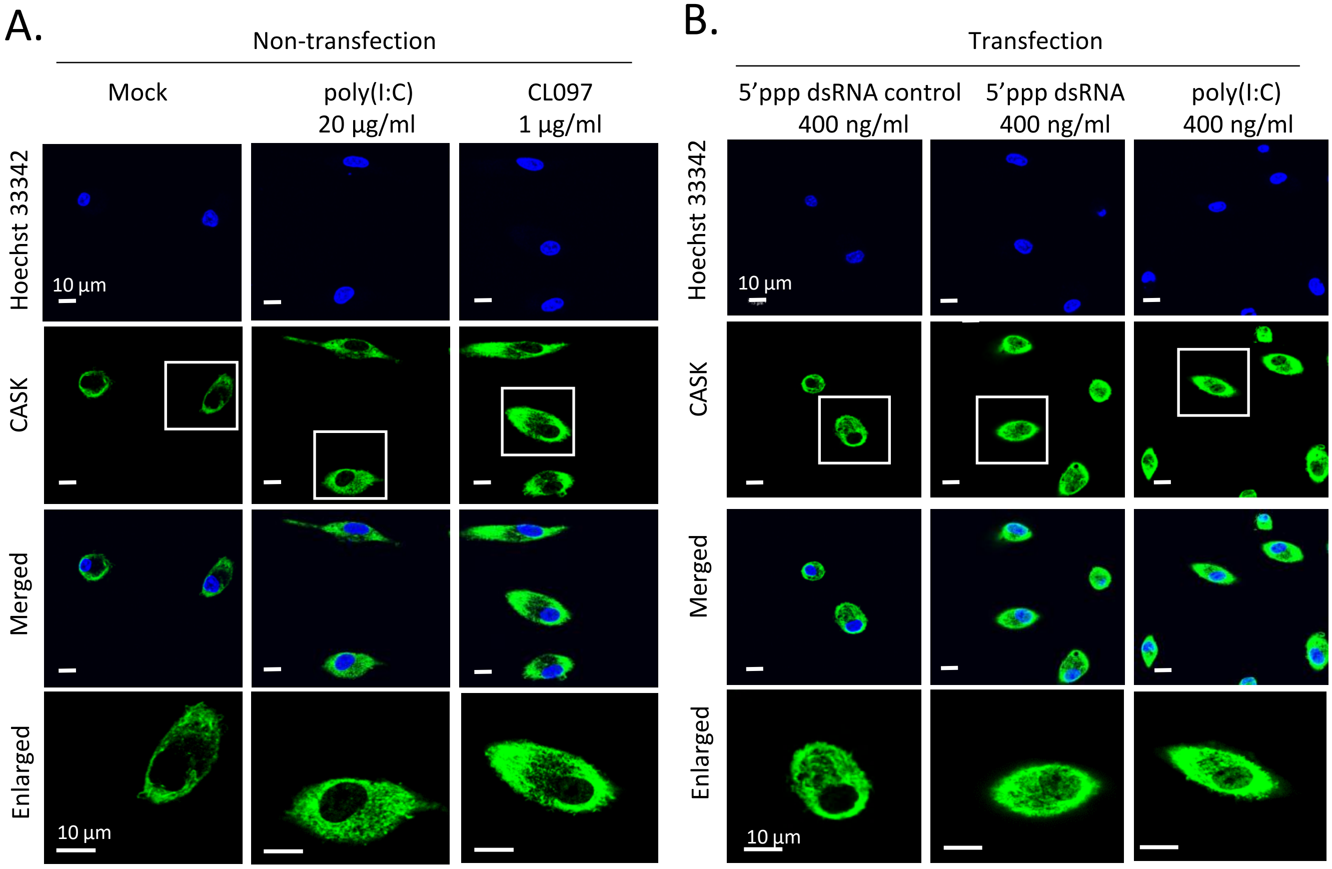

Figure S1 related to Fig.1 Activation of intracellular, but not endosomal, viral RNA recognition receptors, leads to CASK upregulation and nucleus entry. (A-B) CASK subcellular localization in mouse primary GM-macrophage in response to 3-hour treatments analyzed by confocal fluorescent microscope. (A) TLR3 ligand, poly(I:C) 20 µg/ml; TLR7 ligand, Cl097 1 µg/ml. (B) RIG-I ligand, 5'pppRNA 400 ng/ml transfection; MDA-5 ligand, poly(I:C) 400 ng/ml transfection; (A) RIG-I and MDA-5 are intracellular viral RNA recognition receptors, whereas TLR3 and TLR7 are endosomal viral RNA recognition receptors. Scale bar=10 µm.

Figure S2.

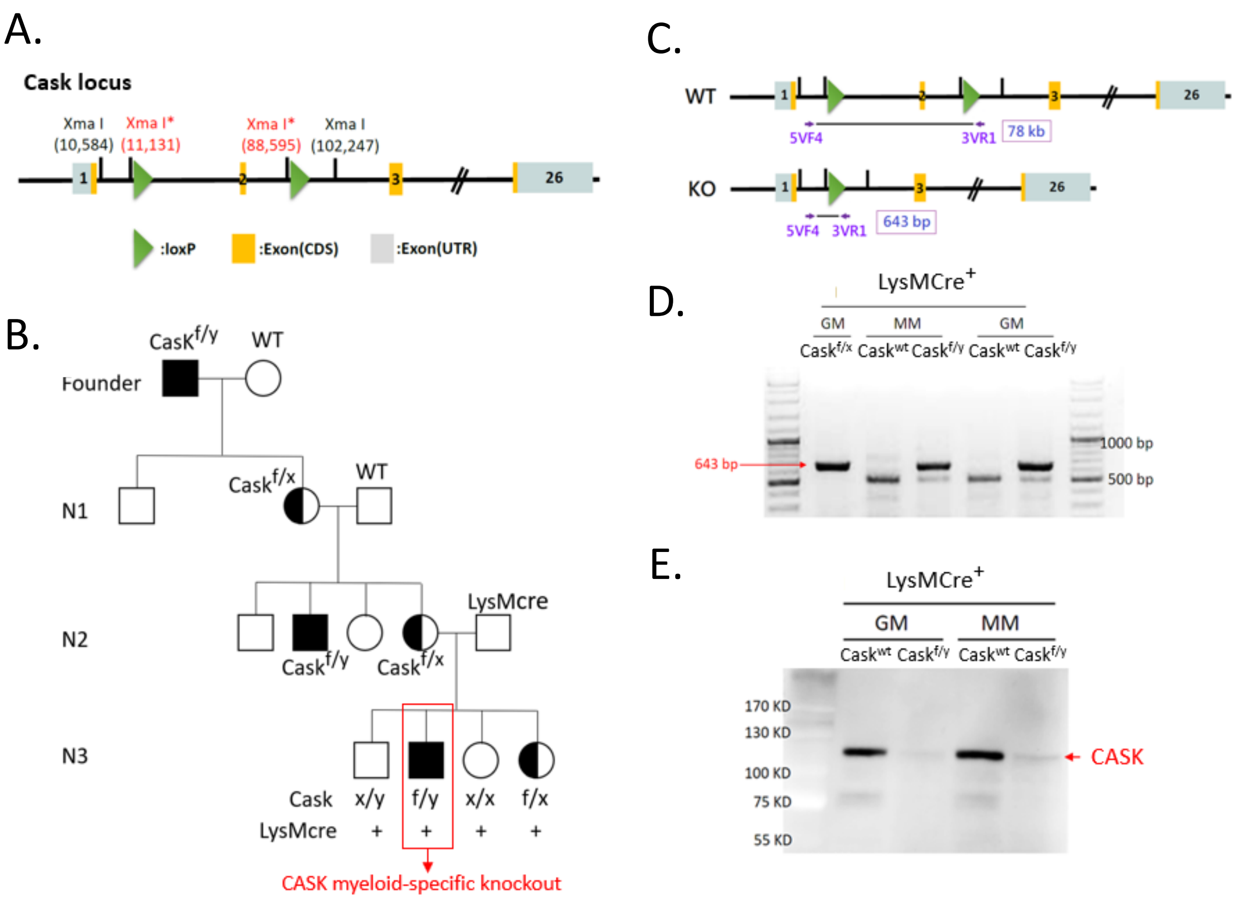

Figure S2 related to Fig.2 Generation of myeloid-specific CASK knockout mice. (A) Insertion of lox-p sequences flanking CASK exon 2 in pronuclei using CRISPR-mediated gene editing approach. (B) Breeding scheme for generating myeloid-specific CASK knockout mice (*Cask*$^{f/y}$LysMcre^{+}). We use N≥4 generations of mice to minimize potential off-target effect. (C) Genotyping primer designed for CASK knockout. The PCR product length is 643 bp for CASK knockout. (D-E) GM, mouse primary GM-macrophage, differentiates in GMCSF-supplemented medium (10 ng/ml) for 7 days; MM, mouse primary MM-macrophage, differentiates in MCSF-supplemented medium (10 ng/ml) for 7 days. (D) Genotyping of CASK locus in mouse primary macrophages derived from *Cask*^{wt}LysMcre^{+} and *Cask*$^{f/y}$LysMcre^{+} mice. The gel band at 643 bp suggests successful removal of exon 2. (E) CASK knockout efficiency check: CASK protein levels in GM- and MM-macrophages derived from *Cask*^{wt}LysMcre^{+} and *Cask*$^{f/y}$LysMcre^{+} mice.

Figure S3.

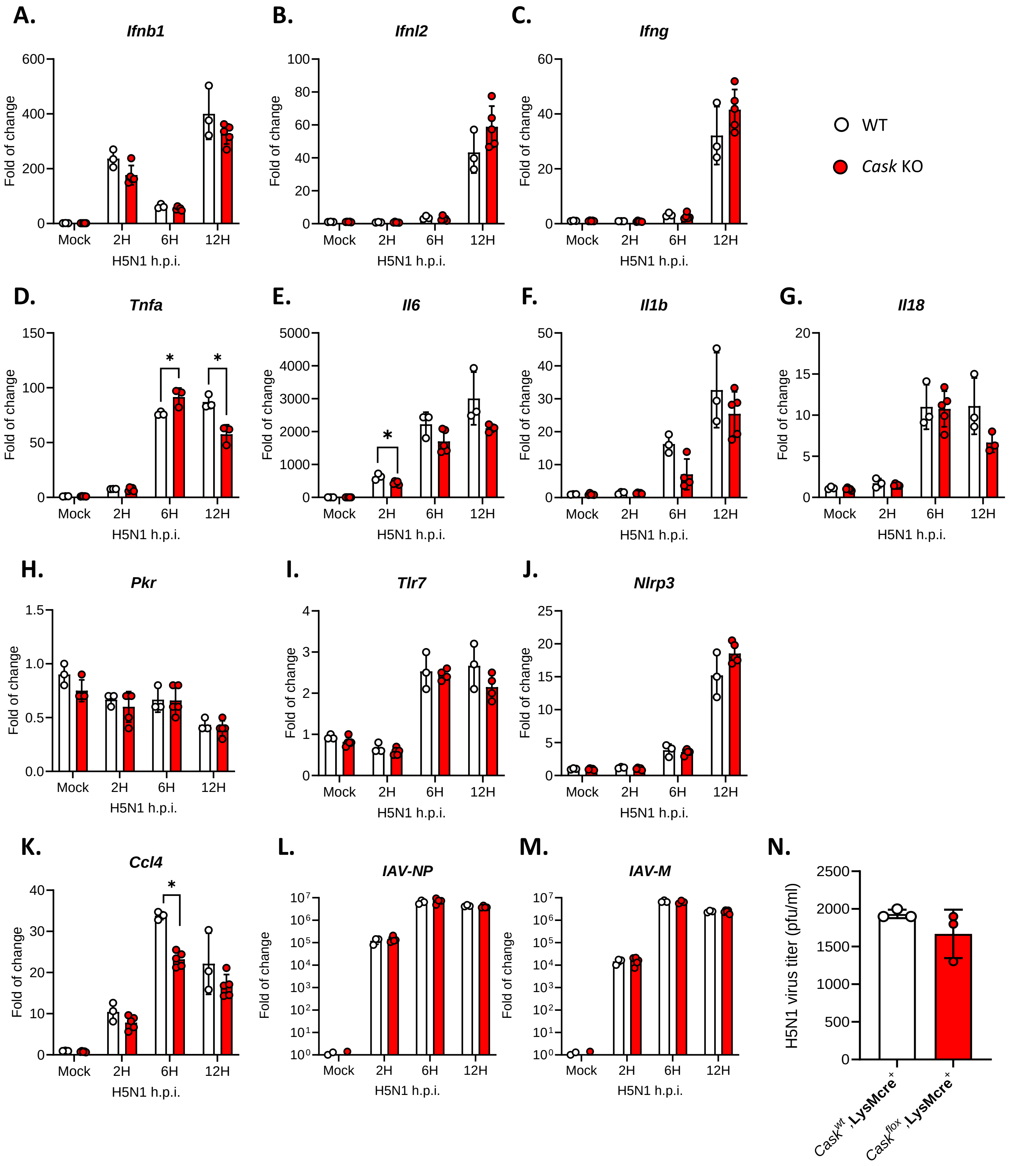
A.
Ifnb1
B.
Ifnl2
C.
Ifng
WT
Cask KO
D.
Tnfa
E.
Il6
F.
Il1b
G.
Il18
H.
Pkr
I.
Tlr7
J.
Nlrp3
K.
Ccl4
L.
IAV-NP
M.
IAV-M
N.
Fold of change
H5N1 h.p.i.
Mock
2H
6H
12H
H5N1 virus titer (pfu/ml)
Cask^wt ;LysMcre^+
Cask^flox ;LysMcre^+

Figure S3. related to Fig.2 The effects of CASK deficiency on transcriptional upregulation of interferons, proinflammatory cytokines, inflammasomes, interferon-stimulated genes, chemokines, viral genes and H5N1 titer, in response to H5N1 infection. infection. (A-M) Mouse primary GM-MΦ are infected with H5N1 (MOI=1) for indicated time periods, and cells are collected for RNA extraction and reverse transcription. mRNA levels of interferon genes are determined by real-time quantitative PCR. WT: $Cask^{wt}$LysMCre+ (white circles), Cask KO: $Cask^{flox}$LysMCre+(red circles). (A-C) Interferons, (A) *Ifnb*, (B) *IfnL2*, (C) *IfnG;* (D-G) proinflammatory cytokines, (D) *Tnfa*, (E) *Il6*, (F) *Il1b,* (G) *Il18;* (H&I) Interferon-stimulated genes, (H) *Pkr*, (I) *Tlr7*. (J) an inflammasome gene, *Nlrp3;* (K) chemokines , *Ccl4;* (L-M) Influenza A virus genes, (L) *NP, (M) M; (N) H5N1 titer measured by plaque assay*.

Figure S4.

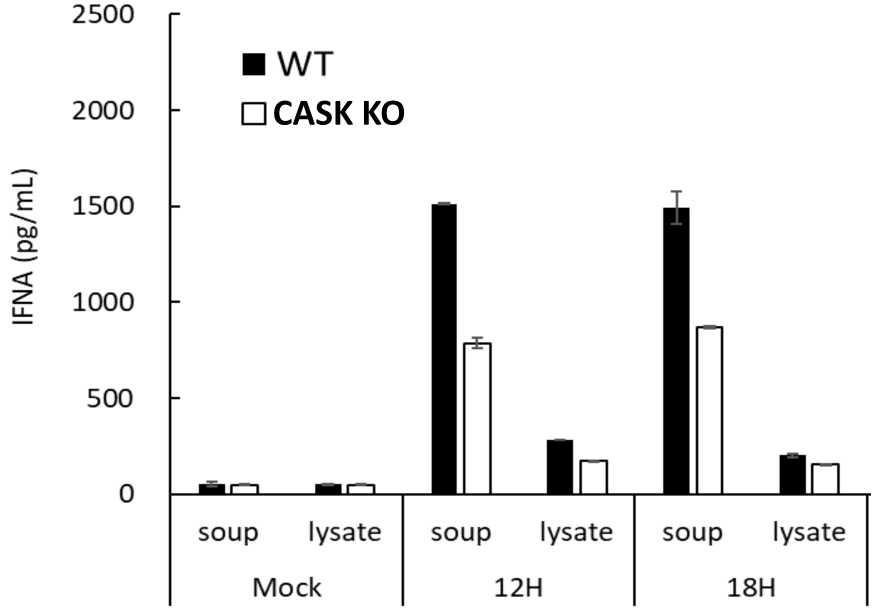

Figure S4. CASK deficiency does not affect IFNA secretion. Mouse primary GM-MΦ were infected with H5N1 at an MOI of 1. At 12 or 18 H (hours)) post-infection, cell culture supernatants and lysates were collected. IFNA levels were quantified by ELISA, with IFNA in the supernatant representing secreted cytokines and IFNA in the lysate indicating intracellular accumulation.
